# Full-length *in meso* structure and mechanism of rat kynurenine 3-monooxygenase inhibition

**DOI:** 10.1038/s42003-021-01666-5

**Published:** 2021-02-04

**Authors:** Shinya Mimasu, Hiroaki Yamagishi, Satoshi Kubo, Mie Kiyohara, Toshihiro Matsuda, Toshiko Yahata, Heather A. Thomson, Christopher D. Hupp, Julie Liu, Takao Okuda, Kenichi Kakefuda

**Affiliations:** 1 Drug Discovery Research, Astellas Pharma. Inc., Tsukuba, Ibaraki Japan; 2 X-Chem Inc., Waltham, MA USA; 3 Present Address: Electronic Materials Department, Idemitsu Kosan Co., Ltd., Sodegaura, Chiba Japan; 4 Present Address: Civetta Therapeutics, Cambridge, MA USA; 5 Present Address: Formulation, Tsukuba Laboratory, Nemoto Science Co., Ltd, Joso-shi, Ibaraki Japan

**Keywords:** X-ray crystallography, Pharmacology, Neurodegeneration, Enzyme mechanisms

## Abstract

The structural mechanisms of single-pass transmembrane enzymes remain elusive. Kynurenine 3-monooxygenase (KMO) is a mitochondrial protein involved in the eukaryotic tryptophan catabolic pathway and is linked to various diseases. Here, we report the mammalian full-length structure of KMO in its membrane-embedded form, complexed with compound **3** (identified internally) and compound **4** (identified via DNA-encoded chemical library screening) at 3.0 Å resolution. Despite predictions suggesting that KMO has two transmembrane domains, we show that KMO is actually a single-pass transmembrane protein, with the other transmembrane domain lying laterally along the membrane, where it forms part of the ligand-binding pocket. Further exploration of compound **3** led to identification of the brain-penetrant compound, **5**. We show that KMO is dimeric, and that mutations at the dimeric interface abolish its activity. These results will provide insight for the drug discovery of additional blood-brain-barrier molecules, and help illuminate the complex biology behind single-pass transmembrane enzymes.

## Abstract (web summary)

Mimasu et al. report a rat full-length structure of kynurenine 3-monooxygenase (KMO) in its membrane-embedded form, complexed with two inhibitors. They find that the dimeric interface of KMO is critical for its activity. This study provides insights into the drug development strategies that target KMO, an important regulator of tryptophan metabolism.

## Introduction

Tryptophan is an essential amino acid in mammals and undergoes degradation through the kynurenine pathway^1–5^. Metabolite imbalances in this pathway are correlated with a wide range of brain disorders^6–9^. Kynurenine 3-monooxygenase (KMO) is a regulator of this pathway and its inhibition leads to improvements in Huntington’s-disease-relevant phenotypes^10–14^, pain^15–17^, pancreatic disorders^1,18^ and cancer^19^. KMO is a class A flavoprotein monooxygenase, which catalyses the hydroxylation of L-kynurenine to 3-hydroxykynurenine (3-HK)^4^. NADPH acts as a cofactor while 3-HK is considered a neurotoxin for neurodegenerative disorders^11,15,16^. Inhibiting KMO reduces 3-HK production, thereby rectifying the imbalance^6,11,18,20,21^.

Previous studies have provided evidence suggesting that the soluble region of KMO is auto-inhibitory in *Homo sapie*ns (Hs-KMO)^22^, *Saccharomyces cerevisiae* (Sc-KMO)^23^, and *Pseudomonas fluorescens* (Pf-KMO)^18,22,24^, identifying key residues and elucidating the mechanism of the hydroxylase reaction. Inhibitory modes of UPF 648^23^, GSK180^18^, and Ro 61-8048^25^ including allosteric sites for Pf-KMO have been elucidated^22^. In terms of pharmacology, a recent study reported a prodrug that showed brain permeability and neuroprotective inhibition of KMO^26^. Despite recent progress, however, the reported KMO structures are not fully functional, and little is known about the architecture of KMO as a membrane protein, especially the C-terminal portion, which is indispensable for catalytic activity in mammals. Furthermore, current KMO inhibitors possess a carboxylic acid group and are thought to be unable to penetrate the blood-brain barrier^18,23,25^. No potent, brain-penetrant inhibitor of KMO has been reported to date. To address these holes in the research, we determined the *in meso* crystal structure of the full-length *Rattus norvegicus* (Rat) KMO.

## Results and discussion

### Structure-activity relationships of KMO inhibitors

We performed a high kynurenine-content enzyme assay for our cell-free assay, and used LC/MS for our cellular assays. Findings from these assays are in good agreement with the previous reports^20,21,27^. Specifically, results from our cell-free assay are in good agreement with an independent report from Jacobs et al., who used 600 μM kynurenine and obtained an IC_50_ value of 0.78 ± 1 μM for Ro 61-8048^27^. We used a high concentration of kynurenine (1200 μM) and obtained a similar IC_50_ value of 1.4 μM (Supplementary Table 1). Ro 61-8048 is a known competitive inhibitor of the substrate kynurenine, and the K_M_ of kynurenine changes upon addition of Ro 61-8048^20^. Therefore, the IC_50_ values of Ro 61-8048 are expected to be higher when kynurenine levels in the system are high. Our cellular IC_50_ of 1.2 μM for Ro 61-8048 is also in good agreement with the value reported by Winkler et al. of 0.64 μM (Supplementary Table 2)^20^.

**Fig. 1:**
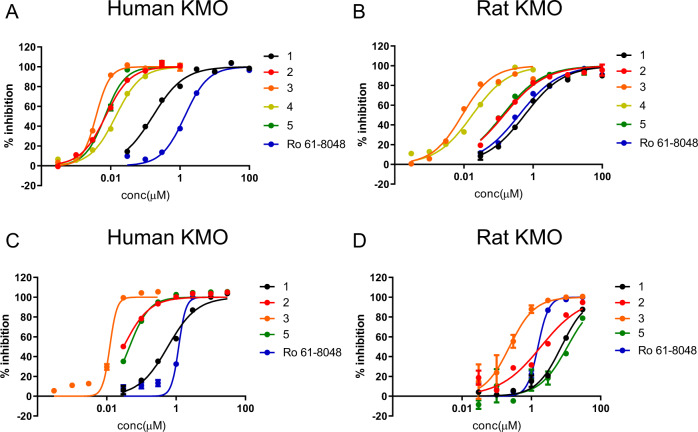
KMO cell-free and cellular assay data. KMO cell-free assay data for **A** Hs-KMO and **B** Rat-KMO. KMO cellular assay data for **C** Hs-KMO and **D** Rat-KMO. Cell-free assays contained high concentrations of the substrate kynurenine and were quantified by detecting absorbance. Cellular assays were measured using LC/MS.

We designed inhibitors to increase potency and aid crystallisation. In our initial investigation, compound **1** was identified as the lead compound with moderate KMO inhibitory activity (KMO IC_50_ = 0.16 µM, 0.57 µM for human, rat, respectively) (Table 1, Fig. 1a–d). Introduction of halogens to the left part of the phenyl group resulted in an increase in KMO inhibitory activity (Table 1, compound **2**), although the species difference in potency remained. Furthermore, introduction of a benzyloxy group at the 2-position of benzoic acid led to a dramatic increase in KMO potency, especially in the rat homolog (Table 1, compound **3**). The potency of compound **3** enabled high resolution crystal structures to be obtained from crystals using in-house screens.

**Table 1:** Chemical structure and structure activity relationship of KMO-inhibiting compounds.

No.	Compound	Human KMO IC_50_ (µM)	Rat KMO IC_50_ (µM)	Human cell-based assay IC_50_ (µM)	Rat cell-based assay IC_50_ (µM)	Calculated pKa
**1**		0.16	0.57	0.58	7.3	4.2
**2**		0.0071	0.15	0.030	1.9	4.1
**3**		0.0038	0.0090	0.012	0.24	3.0
**4**		0.014	0.017	–	–	2.2
**5**		0.0068	0.13	0.046	11	7.2

Compound **1** was identified as our lead compound. Optimisation of **1** produced a potent inhibitory compound, **3**, for which the crystal structure was determined. Compound **4** was identified in a DNA-encoded chemical library screen. Replacing the carboxylic acid moiety of compound **3** yielded compound **5**.

### Crystal structure of full-length Rat-KMO *in meso*

**Fig. 2:**
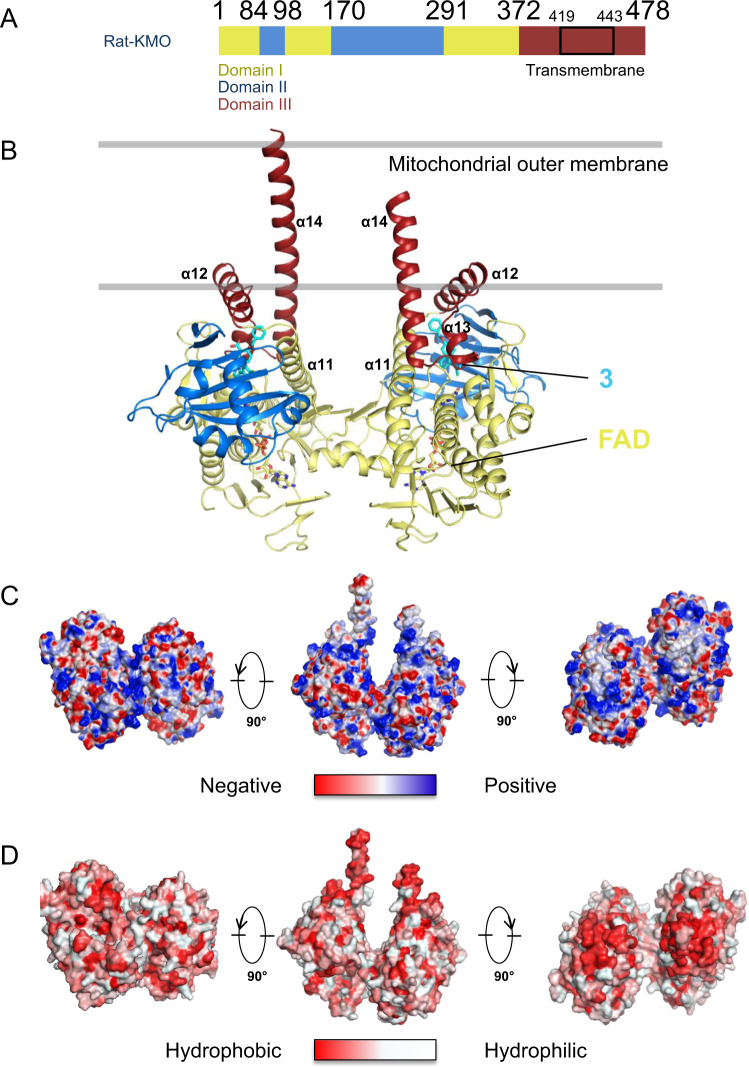
Crystal structure of full-length Rat-KMO *in meso*. **A** Organisation of Rat-KMO domain. **B** The overall structure of Rat-KMO in complex with compound **3** in its membrane-embedded dimeric form. The two predicted transmembrane regions exhibit distinct features. One transmembrane region is inserted into the lipid bilayer while the other lays laterally across the membrane, forming part of the ligand binding pocket. Domains I, II and III are coloured yellow, blue, and red, respectively. Compound **3** and FAD are coloured cyan and yellow, respectively. Location of the membrane is shown in grey, as suggested from analysis of the sequence, electrostatics, and hydrophobicity. **C** Electrostatic potential and **D** hydrophobicity colouring of Rat-KMO show a non-polar surface in the α12 helix, suggesting that it is partially embedded in the membrane.

Hs-KMO is a 56 kDa protein that shares 93% similarity with Rat-KMO (Supplementary Fig. 1). We purified full-length Rat-KMO by expressing Rat-KMO in insect cells using baculovirus and subsequent solubilisation with DDM (Supplementary Fig. 2a, b). We determined the full-length structure of Rat-KMO in its membrane-embedded form complexed with compound **3** and compound **4** at 3.0 Å resolution. Here, we will describe the structure of compound **3** to explain the overall features. The Rat-KMO monomer consists of three domains^28^: α + β flavin adenine dinucleotide (FAD)-binding domain I, FPMO enzyme-conserved domain II containing six-stranded β-sheets, and C-terminal domain III, which is predicted to be comprising two transmembrane domains, although the exact topology remains ambiguous^22,29^. Unexpectedly, the crystal structure showed that the C-terminal region was comprising only one transmembrane domain, with the other predicted transmembrane helix lying laterally along the membrane (Fig. 2a, b, Table 2, Supplementary Figs. 3a, b, c,  4a, b,). This helix displayed hydrophobic residues on the outer side, suggesting that it is partially embedded in the membrane (Fig. 2c, d). This architecture is also found in the σ1 receptor^30,31^. To our surprise, this helix surrounded the benzene ring of compound **3**, which comprises part of the ligand-binding pocket.

**Table 2:** Data collection and refinement statistics (molecular replacement).

	Rat-KMO in complex with compound 3	Rat-KMO in complex with compound 4
*Data collection*		
Space group	C 1 2 1	C 1 2 1
Cell dimensions		
* a*, *b*, *c* (Å)	160.97, 63.43, 152.42	161.51, 63.42, 152.66
α, β, γ (°)	90.0, 113.5, 90.0	90.0, 113.4, 90.0
Resolution (Å)	49.3–3.0 (3.18–3.00)*	140.1–3.0 (3.18–3.00)*
*R*_merge_	0.99 (7.01)	0.83 (9.66)
*CC*_*1/2*_ (%)	95.7 (53.3)	98.5 (51.5)
*I*/σ*I*	8.77(1.2)	8.35 (1.20)
Completeness (%)	99.8 (99.9)	99.8 (99.9)
Redundancy	22.8 (22.7)	27.1 (26.8)
*Refinement*		
Resolution (Å)	49.3–3.0	140.1–3.0
No. reflections	27,209	27,354
*R*_work_/*R*_free_	22.0/26.2	21.8/27.0
No. atoms	6964	6974
Protein	6778	6786
Ligand/ion	180	182
Water	6	6
*B*-factors		
Protein	53.5	72.4
Ligand/ion	34.4	50.0
Water	18.6	46.6
R.m.s. deviations		
Bond length (Å)	0.012	0.012
Bond angle (°)	1.741	1.722

^*^Approximately 300 crystals were used for each structure.

Values in parentheses are for the highest-resolution shell.

To provide further evidence that KMO possesses a transmembrane domain using non-structural methods, KMO liposomes were reconstituted (Supplementary Fig. 5a). Both monomeric and dimeric KMO were observed upon reconstitution, which is consistent with the solved structure. Dimeric KMO was observed in its native state in the presence of SDS and the absence of ligands and cross-linking reagents, suggesting that the dimerisation is fortified by the association between the C-terminal region and the liposome. KMO liposomes retained their catalytic activity, showing that KMO can function in the reconstituted *in meso* state (Supplementary Fig. 5b).

When the structure of Rat-KMO was superimposed with that of the previously determined Hs-KMO, Sc-KMO and Pf-KMO, we observed that the C-terminus domain differed remarkably (Supplementary Fig. 6a). The main chains of Rat-KMO were comparable to that of reported Hs- and Sc-KMO structures at the FAD-binding domain but started to differ at the end of α11. Superimposition of Rat-KMO and Pf-KMO showed partial overlap at α12; however, Pf-KMO turns at the end of α11, which is followed by a bundle of four helices.

### Extended ligand-binding pocket of *in meso* KMO and its binding mode

**Fig. 3:**
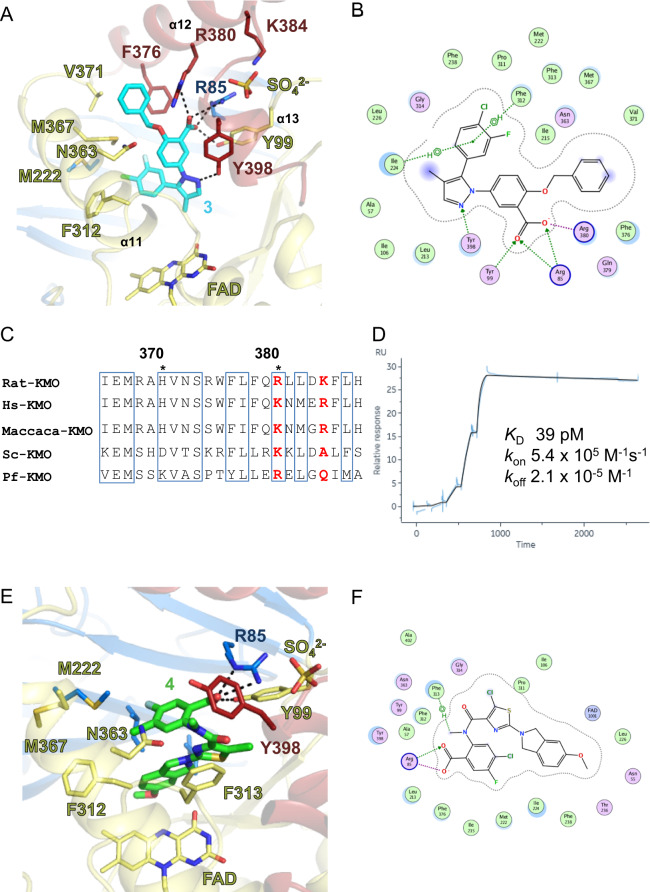
Ligand-binding pocket of full-length Rat-KMO with inhibitors. **A**, **B** Ligand-binding pocket of Rat-KMO inhibited by compound **3**. Residues of α12 play key roles in compound 3 recognition. Key residues for compound **3** recognition are depicted as sticks. Sticks and secondary structures are coloured using the same scheme as that in Fig. 1. Hydrogen bonds are shown as broken lines. Detailed interactions are shown using MOE software. **C** Sequence alignment of the KMO C-terminal residues among species. R380 is K380 in human KMO. Discrepancies in the sequence at this region may explain the species differences observed in our pharmacological assays. **D** Surface plasmon resonance study of compound **3** shows a remarkably low k_off_ value. **E**, **F** Crystal structure of Rat-KMO in complex with compound **4**. Y398 was flipped to accommodate the methyl group and the isoindoline ring of compound **4**, and protruded into a site perpendicular to and above FAD. Key residues and secondary structures are coloured using the same scheme as that in Fig. 1. Compound **4** is shown as green sticks.

The ligand-binding pocket of the full-length Rat-KMO was composed not only of residues from the FAD-binding domain but also of residues from the C-terminal region (Fig. 3a, b). Surprisingly, R380, a residue from this C-terminal region, formed hydrogen bonds with the carboxylic acid moiety of compound **3**. Although Rat-KMO shares 93% sequence similarity with Hs-KMO, and Pf- and Sc-KMO are thought to have a highly conserved ligand-binding pocket, strikingly, the α12 helix residues showed low conservation (Fig. 3c). To visualise the sequence differences, the Hs-KMO model structure was generated using the structure determined in this study (Supplementary Fig. 6b). Five residues in the C-terminal region starting from K380 differed from that in Rat-KMO (Supplementary Fig. 6c). This sequence discrepancy likely underlies the difference in potency between these species, as shown in Table 1.

Compound **3** was recognised by hydrogen bonds on one side by residues R85, Y99, R380 and Y398. On the other side, the chlorofluorophenyl group and benzyloxy group formed hydrophobic interactions with surrounding residues I215, M222, I224, F312, V371, M367, and F376 (Fig. 3a, b). The hydrophobic interactions may explain the remarkably low k_off_ values in surface plasmon resonance experiments (Fig. 3d). No strong contacts between FAD and compound **3** were observed. The structural insights identified in this study are in concert with those of previously reported mutational assays^23,25^. Comparison of the reactive cavity with previously determined KMO structures from other species suggests that the most notable difference is at residues around α11–13 (Supplementary Fig. 7a, b, c). The α12 and α13 helices were not present in the Sc-KMO or Hs-KMO structure. In the Pf-KMO structure, the angles of α11 and α12 helices were different, showing poor overlay. These differences contribute to the change in overall shape and volume of the ligand-binding pocket.

### Docking studies of KMO representative inhibitors

To observe whether this difference alters the inhibitor-binding mode, we performed docking studies of representative KMO inhibitors using the structure determined in this study. First, we validated our structure by docking it with the substrate kynurenine (Supplementary Fig. 8a). The carboxylic acid moiety of kynurenine fit into the polar side of the pocket with R85 and the aniline moiety of kynurenine docked on the hydrophobic side, consistent with evidence that it forms key interactions in previously reported structures and mutational assays^25,27^. Utilising this insight, docked compounds were selected based on whether the polarised or hydrophobic regions of the compounds fit into the respective regions of the KMO pocket. Second, we performed docking studies of representative KMO inhibitors (Supplementary Fig. 8b–d). Key interactions, such as that between the carboxylic acid moiety of the compound and R85, were observed in GSK180 and UPF 648. Additionally, the hydrophobic regions of these compounds fell on the hydrophobic side of KMO, demonstrating a similar binding mode to that with kynurenine. Of note, the extra chemical space in the Rat-KMO structure meant that the dichlorobenzyl moiety of GSK180 and UPF 648 was not perpendicular to FAD, which is different from the previous reports^32^. Our docking studies, therefore, suggest a different binding mode to those experimentally determined for non-mammalian or truncated versions of KMO in a lipid-free environment. We propose that our docking studies are more reflective of the true binding mode in humans given that we examined the full-length and mammalian nature of KMO *in meso*. The extra chemical space between the compound and FAD may be an entry site for NADPH. The binding mode of Ro 61-8048 was determined in a similar manner by taking into account of the polarised and hydrophobic sides of the pocket. Given that reports have shown that Ro 61-8048 binds both to allosteric and canonical binding sites of Pf-KMO, but only to the canonical binding site of Sc-KMO, we speculated that Ro 61-8048 may bind to at least the canonical substrate binding site of mammalian KMO^22^.

Due to the major structural rearrangements of α12 and α14, we were unable to unambiguously identify a secondary allosteric site, as was found in Pf-KMO, in our determined structure (Supplementary Fig. 6a). Helix α12 would have to embed itself deeply into the membrane to form an allosteric pocket, and it is unclear if this structural arrangement is possible in mammalian KMO. All reported KMO allosteric binding sites apply to Pf-KMO only (Supplementary Table 3)^22,25^. Because Hs-KMO was determined only in its autoinhibited state, which lacks the C-terminal domain, it is impractical to speculate on the allosteric site. In Sc-KMO, Ro 61-8048 binds the canonical kynurenine binding site. It is unclear whether the allosteric binding site exists in mammalian KMO; further studies are needed to examine this. Biochemical studies show that Ro 61-8048 is a substrate competitive inhibitor^20^. Given our findings and the fact that we were unable to find a distinct allosteric binding site, we decided to dock Ro 61-8048 at the canonical substrate binding site.

We think the docking poses of kynurenine-inspired inhibitors such as GSK-180 and UPF 648 are reasonable and consistent with previous reports. We carefully selected our poses based on the interactions observed in the distinct polarised and hydrophobic pocket. For Ro 61-8048, the docked structure was a more speculative result which utilised the renewed pocket. Docking studies have limitations because they require the specification of a centroid around a starting compound to restrict the region within which to dock the compound of interest, thus requiring an unambiguous binding site on which to perform the studies. Furthermore, docking studies rely on a static determined structure and therefore do not account for movements of KMO residues.

### Crystal structure of Rat-KMO in complex with a DNA-encoded chemical library hit

A DNA-encoded chemical library screen led to the identification of compound **4**, which showed little structural resemblance to compound **3**, suggesting structural rearrangements in the pocket^33^. The structure of Rat-KMO in complex with compound **4** was determined at 3.0 Å (Fig. 3e, f). Y398 was flipped to accommodate the methyl group and the isoindoline ring of compound **4**, and protruded into a site perpendicular to and above FAD. This interaction would interfere with an incoming NADPH and block the reduction of FAD, which may represent the inhibitory mode of type II KMO inhibitors^29^. Type II inhibitors derived from GSK180 had a pyridyl group protruding from the former dichlorobenzyl moiety. This pyridyl group may fit into the pocket that accommodates the isoindoline ring of compound **4**, which may form an N–H–π interaction with KMO FAD in its reduced form. Such an interaction would stabilise FAD in its reduced FADH_2_ form and prevent electron transfer and the production of H_2_O_2_. M222 and M367 were also flipped to accommodate the chlorofluorophenyl group. No interactions between R380 and the compound were observed, suggesting that R380 is not always a key residue for inhibition.

### Brain-penetrant inhibitor of KMO

Based on our structural analyses, we hypothesised that it would be possible to structurally design a compound with comparable potency by replacing the carboxylic acid group with a bioisostere, because we reasoned that this moiety may underlie the poor brain penetration of compounds **1**–**3**. Therefore, we substituted the carboxylic group with quinazolinedione to obtain compound **5**, which showed remarkable brain penetration of K_p,brain_ = 0.80 (Tables 1, 3). These results show that a brain-penetrant KMO inhibitor with good potency can be designed.

**Table 3:** Pharmacokinetic profile of compound **1** and compound **5**.

	Exposure in plasma and brain		
	Plasma (ng/mL)	Brain (ng/g)	K_p,brain_
**1**	869	25.4	0.029
**5**	7560	6025	0.80

Bioisosteric compound **5** is a brain penetrant inhibitor.

### The functional unit of KMO is dimeric, and dimer interface mutants abolish catalytic activity

**Fig. 4:**
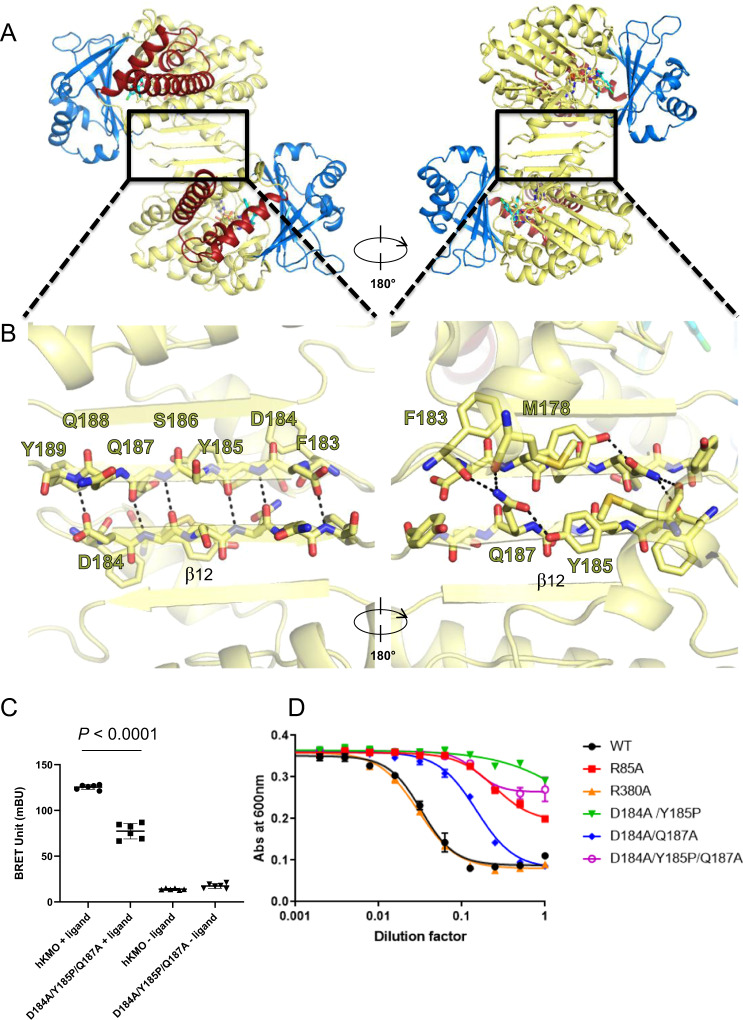
Dimerisation and functional investigation. **A**, **B** Inter-β-sheet hydrogen bonds observed in domain I of Rat-KMO. The crystallographic dimer has 10 hydrogen bonds, in addition to intra hydrogen bonds, linking the dimers. Sticks and secondary structures are coloured using the same scheme as that in Fig. 1. **C** Dimerisation assays for Hs-KMO using NanoBRET. BRET units of non-GST-tagged Hs-KMO and its mutant were measured in HEK293T cells. Mutations at the dimer interface result in significantly lower BRET signals. Error bars represent standard deviation (*n* = 6, *p < 0.0001, two-tailed unpaired Student’s *t*-test). **D** Functional assay of Rat-KMO mutants in mitochondrial membranes. All mutants decreased catalytic activity of Rat-KMO, with activity being almost completely abolished in the presence of the β-sheet-disrupting Y185P mutation. For each sample, the enzyme concentration dilution factor (x-axis) is normalised to the intensity of anti-GST from western blot analyses.

Another intriguing feature of the identified structure of KMO is that it is oriented as a non-antiparallel dimer in the asymmetric unit (Figs. 2b, 4a, b). The KMO monomers are linked by intermolecular β-sheets at F183-Y189 (β12), strongly suggesting physiological dimerisation. This dimer region is conserved in Hs-KMO, Rat-KMO, and Macaca-KMO (Supplementary Fig. 1). Furthermore, reconstituted KMO in liposomes was dimeric (Supplementary Fig. 5a).

To confirm dimerisation at the cellular level, NanoBRET measurements of Hs-KMO were conducted in HEK293T cells (Fig. 4c, Supplementary Fig. 9a). While strong signals of dimerisation were observed in the presence of wild type, a significant decrease in BRET signal was observed in the presence of mutants that weakened the β-sheet dimer interface (D184A, Y185P, and Q187A). The BRET signal of both wild type and mutant KMO was reduced in the absence of the TMR ligand.

To assess the biochemical activity of the dimer interface residues and R380 in the ligand-binding site, mutational assays were conducted using Rat-KMO mitochondrial membranes. The R380A mutation had no effect on kynurenine hydroxylation, suggesting that it does not play a major role in substrate recognition (Fig. 4d, Supplementary Fig. 9b). We then assessed Rat-KMO activity in the presence of mutants that weaken the β-sheet dimer interface, as identified from NanoBRET measurements. Remarkably, all mutants decreased Rat-KMO catalytic activity, and activity was almost completely abolished in the presence of the β-sheet-disrupting Y185P mutation. These results confirm that the functional unit of KMO is a dimer and that mutations at this site diminish its functional activity.

### Conclusion

In conclusion, we determined the full-length mammalian KMO structure in its membrane-embedded form by synthesizing potent inhibitors that bind to KMO. This structure enabled us to identify the potent brain-penetrant compound, **5**. Biochemical and mutational studies informed by the KMO structure confirmed that the physiological unit of KMO is dimeric. Knowledge of this structure is expected to lead to the discovery of KMO drugs for neurological malignancies such as Alzheimer’s disease and to shed light on the mechanism of single-pass membrane enzymes^30,31^.

## Methods

### Protein expression and purification

The Rat-KMO protein for crystallisation was prepared as follows. The GST-tagged full-length KMO (1-478) baculovirus vector contained a thrombin cleavage site inserted between GST and the N-terminus of KMO, and a TEV site followed by a FLAG tag at the C-terminus.

The Rat-KMO protein was expressed using the Bac-to-Bac Baculovirus system in *Spodoptera frugiperda* (Sf9) cells. Cells were infected at a density of 2 × 10^6^ to 3 × 10^6^ cells/mL with a baculovirus multiplicity of infection (MOI) of 0.1, and cultures were grown at 27 °C and collected 2 days after infection.

Cells were then disrupted and solubilised by sonication in Lysis buffer containing 20 mM potassium phosphate buffer pH 7.5, 10% glycerol, 0.5% n-dodecyl β-d-maltoside (DDM), 300 mM NaCl, 7 mM 2-mercaptoethanol and 50 μM FAD supplemented with protease inhibitors. Solubilised Rat-KMO was centrifuged to remove cell debris. The supernatant was pooled and further purified by batch purification using Glutathione Sepharose 4 Fast Flow (GE Healthcare), equilibrated with Lysis buffer. The resin was washed with the same buffer containing 0.012% n-dodecyl β-d-maltoside (DDM), without 50 μM FAD or protease inhibitors. The resin was eluted with 33 mM glutathione. The elution was concentrated with AmiconUltra-15 Centrifugal Filters 50 k (Millipore), and the buffer was subsequently replaced with PD-10 (GE Healthcare) to remove glutathione.

Rat-KMO was digested with thrombin (Novagen) at 10 U/mg at 4 °C overnight. Thrombin was then removed by passing the digested mixture into a Benzamidine Sepharose 6B (GE Healthcare) column. The flow-through fraction was pooled and further passed through Glutathione Sepharose 4 Fast Flow (GE Healthcare). The flow-through fraction was concentrated with AmiconUltra-15 Centrifugal Filters 50 k (Millipore). Concentrated Rat-KMO was then purified using Anti-FLAG M2 Affinity Gel (Sigma-Aldrich) in a buffer containing 20 mM potassium phosphate buffer pH 7.5, 10% glycerol, 0.012% DDM, 300 mM NaCl, 7 mM 2-mercaptoethanol, and eluted with 100 μg/mL FLAG peptide. Eluted Rat-KMO was concentrated with AmiconUltra-15 Centrifugal Filters 50 k (Millipore) and treated with N-GlycosidaseF (Roche) at 10 U/mg at 4 °C overnight. Rat-KMO was further concentrated with AmiconUltra-15 Centrifugal Filters 50 k (Millipore) and gel filtered with Superose 6 10/300 GL (GE Healthcare) in a buffer containing 20 mM potassium phosphate buffer pH 7.5, 10% glycerol, 0.012% DDM, 300 mM NaCl, and 1 mM TCEP. Void fractions were removed and peak elutions were pooled and passed through an Acrodisc Unit with Mustang Q Membrane (0.8 µm 25 mm, Pall Corporation). Rat-KMO was finally concentrated with AmiconUltra-4 Centrifugal Filters 50 k (Millipore) to over 30 mg/mL.

Rat-KMO mutant constructs were generated by PCR using an identical construct to the crystallisation construct but without the TEV and FLAG sites. They were expressed in a similar manner and the membranes were purified using a sucrose gradient of 10–40% in a buffer containing 25 mM HEPES-Na pH 7.5 and 1 mM EDTA.

KMO liposomes were reconstituted by mixing Hs- or Rat-KMO protein with Soybean PC liposomes (L-alpha-phosphatidylcholine, from soybean; Sigma-Aldrich) in PBS solution supplemented with 1% DDM. Detergents were removed by mixing the solution with Bio-Beads SM-2 Adsorbents (Bio-Rad) overnight at 4 °C. Details of the method were established in a previous study^34^. We did not use any cross-linking or additional reagents to facilitate or obtain oligomerisation. The KMO dimer observed in liposomes was in its native state. Protein dimerisation in liposomes confirmed by SDS-PAGE has been observed in other reports^35^.

### Sequence alignment

KMO sequences from *Homo sapiens*, *Macaca mulatta, Rattus norvegicus, Saccharomyces cerevisiae*, and *Pseudomonas fluorescens* were obtained from uniprot O15229 (KMO_HUMAN), F6SV05 (F6SV05_MACMU), O88867 (KMO_RAT), P38169 (KMO_YEAST), Q84HF5 (KMO_PSEFL), respectively, and aligned using Clustal Omega. The aligned sequences were loaded into ESPript 3 (http://espript.ibcp.fr/ESPript/ESPript/) to generate alignments. Secondary structures were manually assigned using the determined structure.

### KMO crystallisation

Rat-KMO was crystallised using the *in meso* method^36^. Purified full-length Rat-KMO was incubated with 4 mM compound **3** and 4 mM NADPH (Roche) at 4 °C for 20 minutes and subsequently centrifuged. The lipidic cubic phase was formed by mixing a 9:1 monoolein:cholesterol (Sigma-Aldrich) molten lipid mixture with a Rat-KMO-compound **3** complex in a coupled syringe at a 2:3 ratio. Crystallisation was performed using Mosquito-LCP (TTP labtech) to dispense 50 nL LCP and 800 nL precipitant into a MRC sitting drop plate (Molecular Dimensions). Crystals of Rat-KMO in complex with compound **3** were precipitated in a reservoir containing 0.04 M Tris-Cl pH 7.0, 0.06 M Bis-Tris pH 6.5, 0.3–0.51 M lithium sulfate, and 34–45% PEG400. Plates were incubated at 17 °C for 1–3 days to control nucleation and then further incubated for crystal growth at 20 °C for at least 10 days.

Crystals in the cubic phase were harvested directly from drops. For crystals formed in the sponge phase, the phase was solidified with a buffer containing 0.04 M Tris-Cl pH 7.0, 0.06 M Bis-Tris pH 6.5, 0.4 M lithium sulfate, and 20–30% PEG400 before the crystals were directly harvested. X-ray diffraction experiments were performed at SPring-8 BL32XU. The wavelength was 1.0000 Å. ZOO^37^ and SHIKA modules were implemented to automatically collect data at 5–7 wedges. The data were processed by XDS^38^, and BLEND in CCP4^39^, using KAMO^40^ software. Over 300 datasets from approximately the same number of crystals were used to obtain a full data set.

Crystals of Rat-KMO in complex with compound **4** were obtained in a similar manner except that NADPH was not used and a precipitating buffer containing 0.04–0.05 M sodium citrate pH 6.5 or 0.04 M Bis-Tris pH 6.5, 0.05–0.06 M Tris-Cl pH 7.0, 0.12–0.43 M lithium sulfate, and 34–46% PEG400 was used. The plates were incubated at 20 °C or 23 °C.

### Rat-KMO modelling structural analysis

Initial phases were solved by molecular replacement with Phaser^41^ using a Rat KMO model derived from Sc-KMO (PDBID:4J34) and Pf-KMO (PDBID:5MZC, 5FN0). The model and phases were refined using COOT and CCP4. Compound **3** was assigned by searching *F*o-*F*c electron densities using AFITT (Openeye), which also generates cif files for refinement. The compound was manually inspected and accordingly refined with COOT^42^ and REFMAC. The C-terminus was disordered from H443. NADPH was included in the Rat-KMO-compound **3** crystallisation drop; however, its resolution could not be observed. The sulfate atom in the structure was placed based on the clear *F*o-*F*c electron density at 5 σ, the interaction of surrounding residues, and the high concentration of sulfate in the crystallisation condition (Supplementary Fig. 4b). However, the presence of other cofactors or ions cannot be excluded. Structures of compound **4** were determined by molecular replacement using the Rat-KMO-compound **3** structure, and its ligands were determined in a similar manner.

All structures were inspected to ensure they suitably fit the Ramachandran plot. The structures had over 95% of residues in the favoured region, less than 5% in the additional allowed region, and less than 0.5% in the outlier region.

Superimposition of Rat-KMO with previously determined structures was performed using Coot. KMO structures from Human (Hs) (PDBID:5X68), *Saccharomyces cerevisiae* (Sc) (PDBID:4J34) and *Pseudomonas fluorescens* (Pf) (PDBID:5MZC) were superimposed. 4J34 was used for Sc-KMO because the disordered C-terminal His-tag was cleaved and determined at the highest resolution.

### Ligand docking and human KMO model construction

Ligands were docked using Schrodinger Maestro software (Schrödinger). Rat-KMO in complex with compound **3** was selected for docking because its side chains resembled previously reported structures. The PDB file was prepared using the protein preparation. The compound **3** binding site was used as the centroid for generating the ligand-binding grid. Ligands were prepared using Ligprep and docked using GLIDE.

Docked compounds were selected based on whether the polarised or hydrophobic regions of the compounds fit into the respective regions of the KMO pocket. The carboxylic acid moiety of the compound interacts with polarised KMO residues such as R85, and the hydrophobic regions of the compounds fit into the hydrophobic sides of the KMO pocket. All docked structures were among the top 5 docked sites, and kynurenine was the top ranked structure.

### Human and rat KMO enzyme assay

All information relevant to compound synthesis of compounds **1–5** are in Supplementary Note 1. Human KMO was purchased from Origene and used to assay compounds. Full-length Rat-KMO purified for crystallisation was used to assay compounds. Full-length Rat-KMO-expressing membranes were used for mutant assays (Fig. 4b) only. Mutant Rat-KMO expressed in membranes was quantified by western blotting using anti-GST (Anti-GST-tag pAb (MBL Code No.PM013)) and *IRDye* 800CW Goat anti-Rabbit (LI-COR Biosciences Cat# *926-32211*) antibodies (Supplementary Table 4). Images were taken using Odyssey CLX (LI-COR Biosciences) and quantified using ImageStudio2.0 (LI-COR Biosciences) software.

Test compounds were dissolved in DMSO and diluted with assay buffer containing 50 mM HEPES, 2 mM MgCl_2_, 0.5 mM EGTA, 1 mM DTT, 0.05% Tween20 and 0.005% of antiform SI (pH = about 7.9) to prepare a 4% DMSO solution. Five microliters of the solution was mixed with 5 μL of enzyme solution (human KMO: 4.5 ng/μL, rat KMO: 4 ng/μL) in a 384-well clear flat bottom plate (Greiner Bio-One, 781101). After 5 min of pre-incubation, reactions were started by adding 10 μL of substrate solution containing 2.4 mM kynurenine and 0.6 mM NADPH. Reactions were run for 90 min at 30 °C, then terminated by adding 5 μL of stop buffer (40 mM maleimide, 2 mM WST-9 and 0.1 mM 1-methoky PMS in water). NADPH consumption, as an indicator of enzyme activity, was detected by measuring the absorbance of WST-9 formazan dye at 600 nm using a plate reader (SPECTRAmax PLUS 384; Molecular Devices). Liposomal assays were performed in the same manner except that the composition of the assay buffer was changed (phosphate-buffered saline, 2 mM MgCl_2_, 1 mM DTT, 0.05% Tween 20).

### Cell-based human and rat KMO inhibition assay

HEK293 cells stably expressing human or rat KMO were maintained in DMEM containing 10% fetal bovine serum, 50 units/mL penicillin, 50 μg/mL streptomycin and 0.2 mg⁄mL zeocine. Cells were seeded at a density of 3.5 × 10^5^ cells/80 μL/well in poly-D-lysine-coated 384-well plates and cultured in a CO_2_ incubator. After 2 overnights, the medium was replaced with 10 μL/well of assay buffer (1 × HBSS containing 20 mM HEPES). Ten microliters per well of the diluted test compounds (final, 0–30 μM, 0.3% DMSO) were added to the respective wells. After ~30 min of pre-incubation at 37 °C, reactions were started by adding 20 μL of substrate solution containing 0.2 mM kynurenine. Reactions were run for 2 h at 37 °C, the assay buffer was discarded and the plate was immediately frozen and stored at −30 °C until LC/MS analysis.

### LC-MS/MS analysis

An 80 µL volume of cold acetonitrile with 2% formic acid was added to the cell pellet in a 384-well plate and centrifuged at 3000 rpm for 10 min at 4 °C. A 40 µL volume of the supernatant was transferred to a new 384-well plate and evaporated to dryness under nitrogen gas at 45 °C. The residue was reconstituted in 100 µL of acetonitrile/water (10:90, v/v) with 0.1% formic acid, and 1 µL was injected into the LC-MS/MS system.

LC/MS/MS was performed on a Shimadzu UFLC Nexera X2 (Shimadzu Corporation, Japan) and an QTRAP® 5500 LC-MS/MS System (SCIEX, Framingham, USA). Chromatographic separation was performed using an Allure PFP Propyl analytical column (5 µm, 2.1 × 100 mm) maintained at 50 °C. The autosampler temperature was set at 15 °C. The mobile phase consisted of formic acid/water/acetonitrile/methanol (0.5/500/250/250, v/v/v/v) and the flow rate was set at 0.3 mL/min. 3-hydroxykynurenine was eluted from the LC column at a retention time of 1.2 min. The MS system was operated using electrospray ionization (ESI) in positive mode. The optimum conditions were curtain gas, 20 psi; ion spray voltage, 5500 V; heated nebulizer temperature (TEM), 700 °C; nebulizing gas (GS1), 70 psi; and heater gas (GS2), 70 psi. Data were acquired in the multiple reaction monitoring (MRM) transition mode, which was 225.0 → 109.9 for the analyte and 228.0 → 109.9 for the internal standard. Analyst® software (SCIEX, Framingham, USA) was used for system control and MultiQuant™ Software (SCIEX, Framingham, USA) was used for data analysis.

### Pharmacokinetic studies using rats

Brain penetration studies were conducted in non-fasted male SD rats [Sprague-Dawley rats Crl:CD(SD), 8 weeks of age]. Compound 1 was intraperitoneally (ip) administered at 10 mg/kg in a solution of saline containing 10% dimethylformamide, 10% propylene glycol, and 4% 1 normality (N) sodium hydrate. Compound **5** was ip administered at 10 mg/kg in a solution of saline containing 17% propylene glycol, 8% HCO-40, 4% Tween80, and 0.3% 1 N HCl aqueous solution. Blood and brain were collected at 1 h after a single administration. The test compound in plasma samples or brain samples was extracted by deproteination with acetonitrile, and then analysed using LC-MS/MS.

### Surface plasmon resonance

Surface plasmon resonance experiments were performed using a Biacore 8K+ (GE Healthcare) instrument. A 20-μg/mL concentration of hKMO (Origene) was immobilised on a Series S Sensor Chip CM5 (GE Healthcare) by NHS/EDC amine coupling. The Rmax was 13,500 resonance units (RU) upon binding.

Five-fold serial dilutions of 50 nM compound **3** finalised in 2% DMSO were made, and run in a buffer containing HBS-P+ (GE Healthcare), 1 mM DTT, and 2% DMSO using single-cycle kinetics.

### NanoBRET assay

Wild type or mutant human KMO was cloned into a pFN21A HaloTag CMV Flexi Vector (Promega) and a pFN31K NLuc CMV-neo Flexi Vector (Promega). Cloned vectors were transfected into HEK293T cells using lipofectamine LTX (Invitrogen) and assayed according to the NanoBRET Nano-Glo Detection System (Promega) assay protocol. Briefly, transfected cells were divided into two pools with or without the HaloTag NanoBRET 618 Ligand and incubated overnight. NanoBRET Nano-Glo substrate was added and immediately detected using an Envision plate reader (PerkinElmer) at donor emission (460 nm) and acceptor emission (620 nm). Cell viability in the plates was determined by measuring luminescence using a CellTiter-Glo 2.0 Assay kit (Promega).

### Statistics and reproducibility

No statistical methods were used unless otherwise stated. The experiments were not randomised and investigators were not blinded during the experiments or outcome assessment.

Compound assays were analysed and plotted on graphs using Prism (GraphPad Software Inc. San Diego, CA, USA). The IC_50_ value of compounds was calculated by defining the inhibition rate in the absence of the enzyme (enzyme assay) or substrate (cell-based assay) as 100% and that in the absence of the test compound and the enzyme as 0%, using a logistic method.

Surface plasmon resonance data were analysed using Biacore Insight Evaluation software. All fits used a simple 1:1 Langmuir binding model showing reasonable fit and low fit *χ*^2^ values.

NanoBRET data were obtained in triplicate from two individual experiments (*n* = 6). Standard deviation (s.d.) was calculated and a two-tailed unpaired Student’s *t*-test was performed using GraphPad Prism software (GraphPad Software, La Jolla, CA, USA).

### Ethical regulations

In pharmacokinetic studies using rats, all animal experimental procedures were approved by the Institutional Animal Care and Use Committee of Astellas Pharma Inc. Furthermore, Astellas Pharma Inc., Tsukuba Research Centre, has been awarded Accreditation Status by The Association for Assessment and Accreditation of Laboratory Animal Care (AAALAC) International.

### Reporting summary

Further information on research design is available in the Nature Research Reporting Summary linked to this article.

## Supplementary information

Supplementary Information

Description of Additional Supplementary Files

Supplementary Data 1

Supplementary Data 2

Reporting Summary

## Data Availability

The Source data underlying Figs. 1a–d, 4c, d, Supplementary Figs. 5b, 9a and Supplementary Fig. 2a are provided as Supplementary Data 1 and 2, respectively. Uncropped versions of gels and blots presented in the Supplementary Figs. 2b, 5a and 9b are available in the Supplementary information. PDB accession numbers 6LKD and 6LKE for Rat-KMO in complex with compound **3** and compound **4** have been deposited, respectively. All relevant data are available from the corresponding author on reasonable request.
