# Supplementary material for: Full-length in meso structure and mechanism of rat kynurenine 3-monooxygenase inhibition
Source: Commun Biol. 2021 Feb 4;4:159. doi: 10.1038/s42003-021-01666-5 (PMC7862291; doi:10.1038/s42003-021-01666-5)
Supplement: Supplementary file 1 — Supplementary Information [file 42003_2021_1666_MOESM1_ESM.pdf]

Supplementary Information

Full-length *in meso* structure and mechanism of rat kynurenine 3-monooxygenase inhibition

Shinya Mimasu, Hiroaki Yamagishi, Satoshi Kubo, Mie Kiyohara, Toshihiro Matsuda, Toshiko Yahata, Heather A. Thomson, Christopher D. Hupp, Julie Liu, Takao Okuda & Kenichi Kakefuda

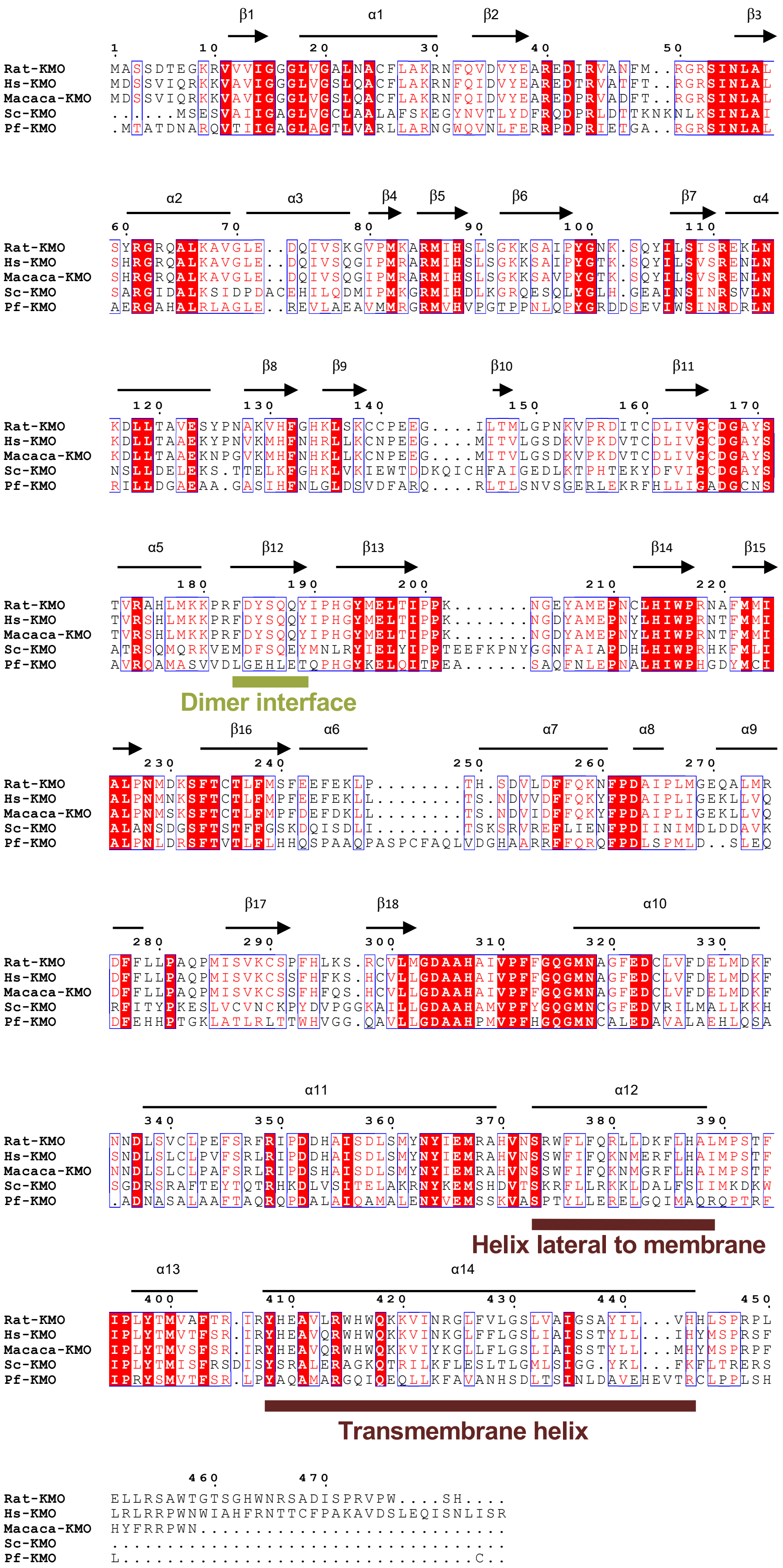

β1 α1 β2 β3
1 10 20 30 40 50
Rat-KMO MASSDTEGKRVVVIGGGLVGALNACFLAKRNFQVDVYEAREDIRVANFM..RGRSINLAL
Hs-KMO MDSSVIQRKKVAVIGGGLVGSLQACFLAKRNFQIDVYEAREDTRVATFT..RGRSINLAL
Macaca-KMO MDSSVIQRKKVAVIGGGLVGSLQACFLAKRNFQIDVYEAREDPRVADFT..RGRSINLAL
Sc-KMO .....MSESVAIIGAGLVGCLAALAFSKEGYNVTLYDFRQDPRLDTTKNKNLKSINLAI
Pf-KMO .MTATDNARQVTIIGAGLAGTLVARLLARNGWQVNLFERRPDPRIETGA..RGRSINLAL
α2 α3 β4 β5 β6 β7 α4
60 70 80 90 100 110
Rat-KMO SYRGRQALKAVGLE..DQIVSKGVPMKARMIHSLSGKKSAIPYGNK.SQYILSISREKLN
Hs-KMO SHRGRQALKAVGLE..DQIVSQGIPMRARMIHSLSGKKSAIPYGTK.SQYILSVSRENLN
Macaca-KMO SHRGRQALKAVGLE..DQIVSQGIPMRARMIHSLSGKKSAVPYGTK.SQYILSVSRENLN
Sc-KMO SARGIDALKSIDPDACEHILQDMIPMKGRMIHDLKGRQESQLYGLH.GEAINSINRSVLN
Pf-KMO AERGAHALRLAGLE..REVLAEAVMMRGRMVHVPGTPPNLQPYGRDDSEVIWSINRDRLN
β8 β9 β10 β11
120 130 140 150 160 170
Rat-KMO KDLLTAVESYPNAKVHFGHKLSKCCPEEG....ILTMLGPNKVPRDITCDLIVGCDGAYS
Hs-KMO KDLLTAAEKYPNVKMHFNHRLLKCNPEEG....MITVLGSDKVPKDVTCDLIVGCDGAYS
Macaca-KMO KDLLTAAEKNPGVKMHFNHKLLKCNPEEG....MITVLGSDKVPKDVTCDLIVGCDGAYS
Sc-KMO NSLLDELEKS.TTELKFGHKLVKIEWTDDKQICHFAIGEDLKTPHTEKYDFVIGCDGAYS
Pf-KMO RILLDGAEAA.GASIHFNLGLDSVDFARQ....RLTLSNVSGERLEKRFHLLIGADGCNS
α5 β12 β13 β14 β15
180 190 200 210 220
Rat-KMO TVRAHLMKKPRFDYSQQYIPHGYMELTIPPK.......NGEYAMEPNCLHIWPRNAFMMI
Hs-KMO TVRSHLMKKPRFDYSQQYIPHGYMELTIPPK.......NGDYAMEPNYLHIWPRNTFMMI
Macaca-KMO TVRSHLMKKPRFDYSQQYIPHGYMELTIPPK.......NGDYAMEPNYLHIWPRNTFMMI
Sc-KMO ATRSQMQRKVEMDFSQEYMNLRYIELYIPPTEEFKPNYGGNFAIAPDHLHIWPRHKFMLI
Pf-KMO AVRQAMASVVDLGEHLETQPHGYKELQITPEA......SAQFNLEPNALHIWPHGDYMCI
Dimer interface
β16 α6 α7 α8 α9
230 240 250 260 270
Rat-KMO ALPNMDKSFTCTLFMSFEEFEKLP........TH.SDVLDFFQKNFPDAIPLMGEQALMR
Hs-KMO ALPNMNKSFTCTLFMPFEEFEKLL........TS.NDVVDFFQKYFPDAIPLIGEKLLVQ
Macaca-KMO ALPNMSKSFTCTLFMPFDEFDKLL........TS.NDVIDFFQKYFPDAIPLIGEKLLVQ
Sc-KMO ALANSDGSFTSTFFGSKDQISDLI........TSKSRVREFLIENFPDIINIMDLDDAVK
Pf-KMO ALPNLDRSFTVTLFLHHQSPAAQPASPCFAQLVDGHAARRFFQRQFPDLSPMLD..SLEQ
β17 β18 α10
280 290 300 310 320 330
Rat-KMO DFFLLPAQPMISVKCSPFHLKS.RCVLMGDAAHAIVPFFGQGMNAGFEDCLVFDELMDKF
Hs-KMO DFFLLPAQPMISVKCSSFHFKS.HCVLLGDAAHAIVPFFGQGMNAGFEDCLVFDELMDKF
Macaca-KMO DFFLLPAQPMISVKCSSFHFQS.HCVLLGDAAHAIVPFFGQGMNAGFEDCLVFDELMDKF
Sc-KMO RFITYPKESLVCVNCKPYDVPGGKAILLGDAAHAMVPFYGQGMNCGFEDVRILMALLKKH
Pf-KMO DFEHHPTGKLATLRLTTWHVGG.QAVLLGDAAHPMVPFHGQGMNCALEDAVALAEHLQSA
α11 α12
340 350 360 370 380 390
Rat-KMO NNDLSVCLPEFSRFRIPDDHAISDLSMYNYIEMRAHVNSRWFLFQRLLDKFLHALMPSTF
Hs-KMO SNDLSLCLPVFSRLRIPDDHAISDLSMYNYIEMRAHVNSSWFIFQKNMERFLHAIMPSTF
Macaca-KMO NNDLSLCLPAFSRLRIPDSHAISDLSMYNYIEMRAHVNSSWFIFQKNMGRFLHAIMPSTF
Sc-KMO SGDRSRAFTEYTQTRHKDLVSITELAKRNYKEMSHDVTSKRFLLRKKLDALFSIIMKDKW
Pf-KMO .ADNASALAAFTAQRQPDALAIQAMALENYVEMSSKVASPTYLLERELGQIMAQRQPTRF
Helix lateral to membrane
α13 α14
400 410 420 430 440 450
Rat-KMO IPLYTMVAFTR.IRYHEAVLRWHWQKKVINRGLFVLGSLVAIGSAYIL...VHHLSPRPL
Hs-KMO IPLYTMVTFSR.IRYHEAVQRWHWQKKVINKGLFFLGSLIAISSTYLL...IHYMSPRSF
Macaca-KMO IPLYTMVSFSR.IRYHEAVQRWHWQKKVIYKGLLFLGSLIAISSTYLL...MHYMSPRPF
Sc-KMO IPLYTMISFRSDISYSRALERAGKQTRILKFLESLTLGMLSIGG.YKL...FKFLTRERS
Pf-KMO IPRYSMVTFSR.LPYAQAMARGQIQEQLLKFAVANHSDLTSINLDAVEHEVTRCLPPLSH
Transmembrane helix
460 470
Rat-KMO ELLRSAWTGTSGHWNRSADISPRVPW....SH....
Hs-KMO LRLRRPWNWIAHFRNTTCFPAKAVDSLEQISNLISR
Macaca-KMO HYFRRPWN............................
Sc-KMO ....
Pf-KMO L...........................C..

Supplementary Figure 1: Sequence alignment of Rat-KMO, Hs-KMO, Macaca-KMO, Sc-KMO and Pf-KMO. Secondary structures were assigned using the structure determined in this study.

A

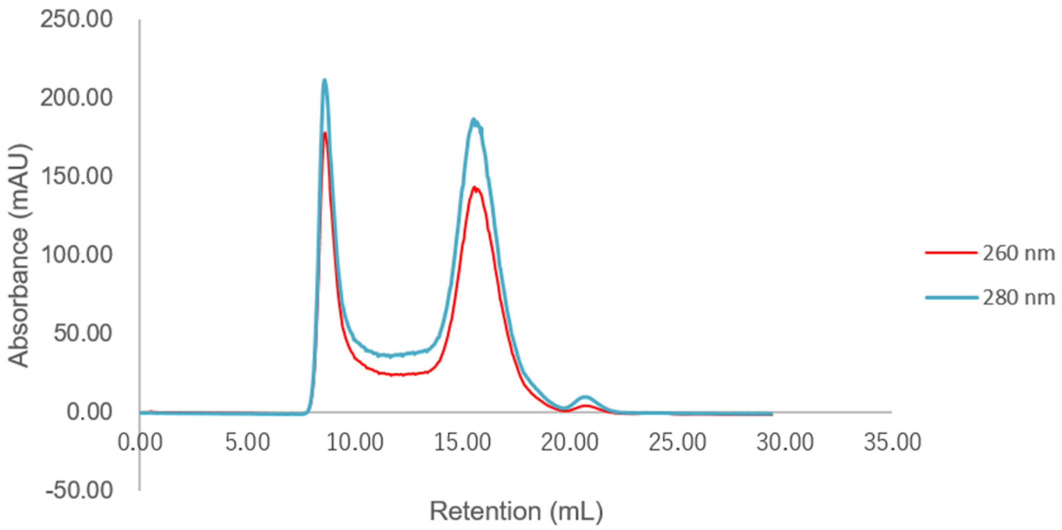

B

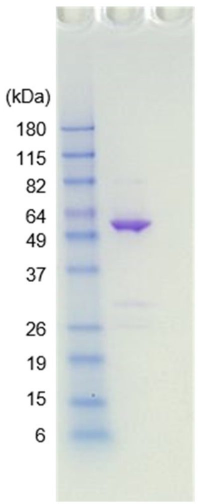

Supplementary Figure 2: Purification of Rat-KMO. (A) Final step: gel filtration chromatography, and (B) SDS-PAGE of the purified protein.

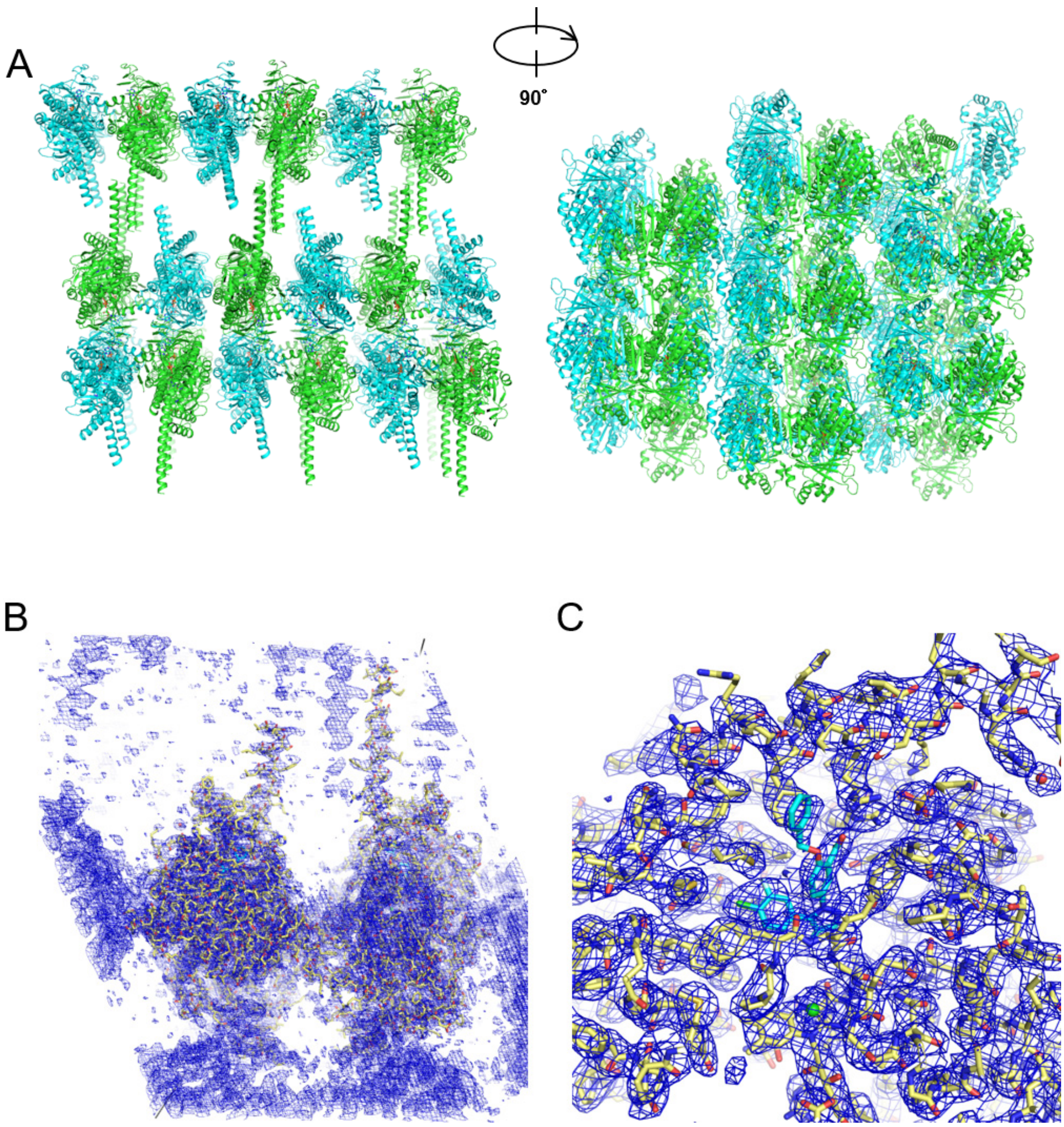

Supplementary Figure 3: Crystal packing and electron density maps of Rat-KMO in complex with compound **3**. (A) Crystal packing of Rat-KMO in complex with compound **3.** Chain A and B are coloured green and cyan, respectively. Two molecules were observed per asymmetric unit. (B) 2 |*F*o| - |*F*c| map showing the overall map of Rat-KMO in complex with compound **3** contoured at 1.0 σ. (C) 2 |*F*o| - |*F*c| map

showing the catalytic pocket of Rat-KMO in complex with compound **3** contoured at 1.3 σ.

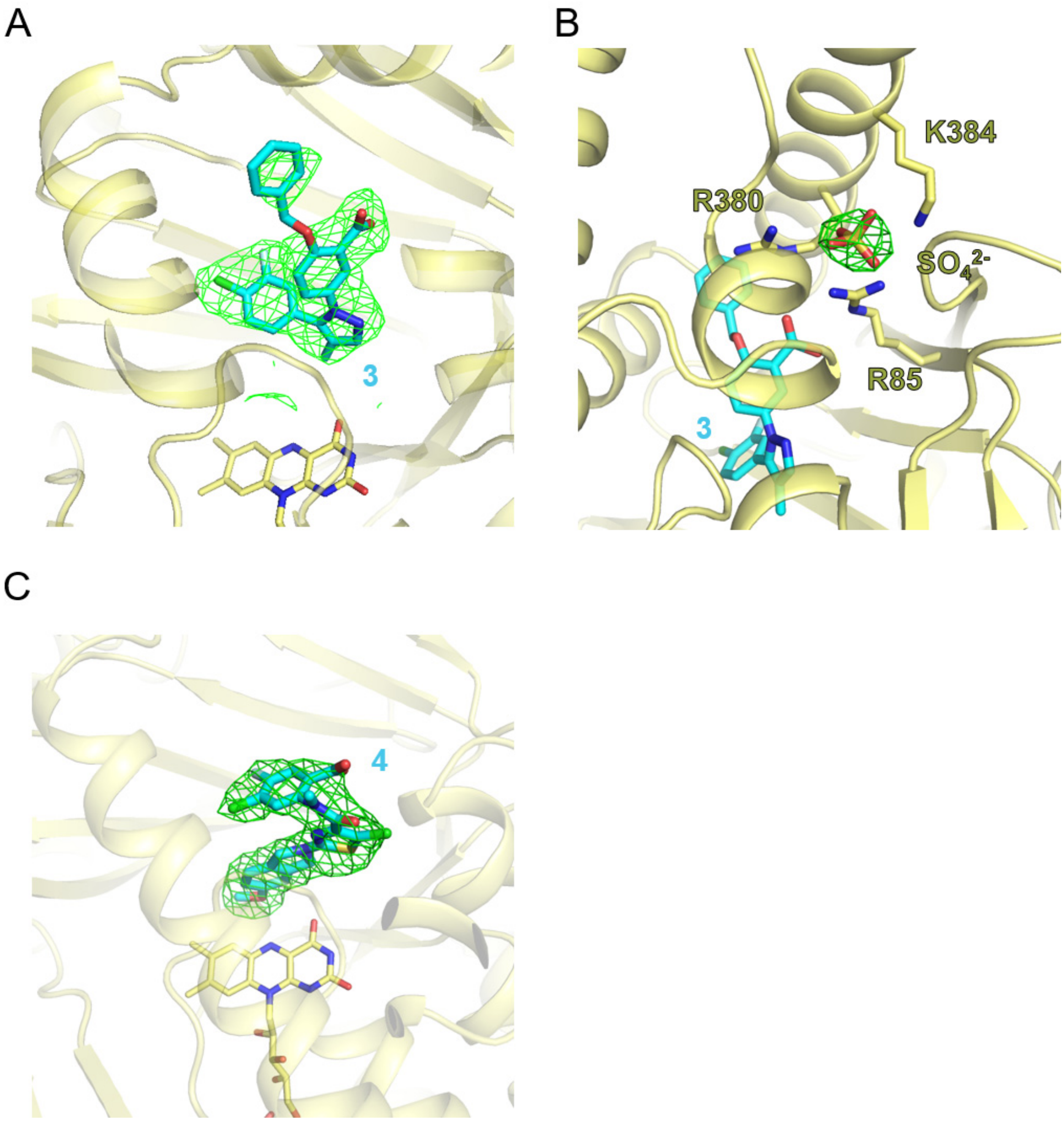

Supplementary Figure 4: |*F*o| - |*F*c| omit maps of (A) compound **3**, (B) sulphate ion and (C) compound **4** contoured at 2.7 σ, 5.0 σ, and 3.0 σ, respectively.

A

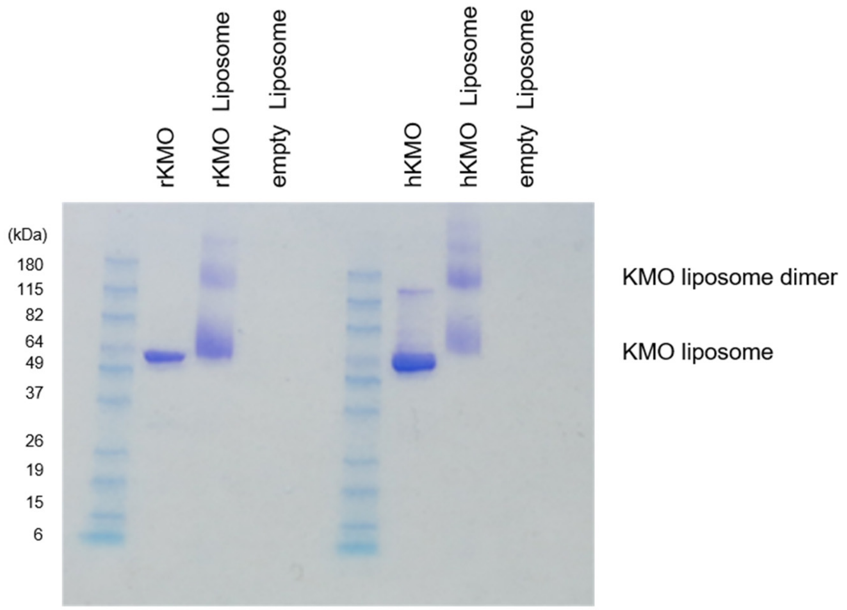

B

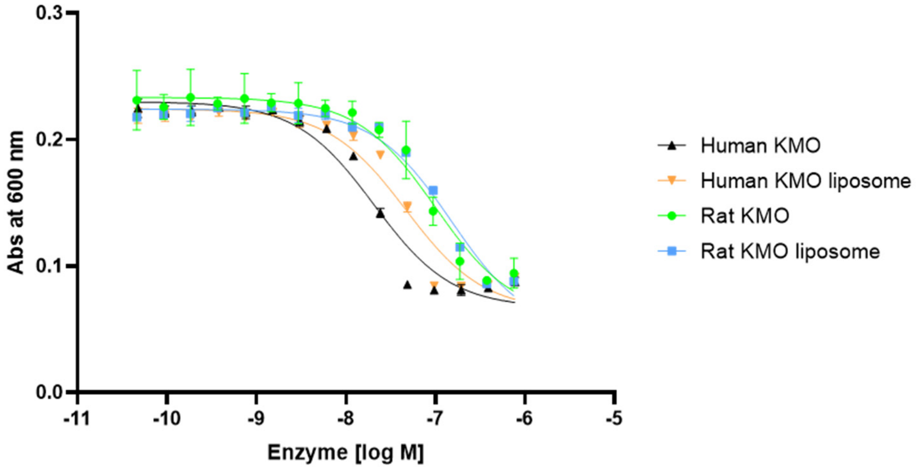

Supplementary Figure 5: KMO liposome reconstitution and catalytic activity. (A) Liposome reconstitution of Hs-KMO and Rat-KMO. Hs- and Rat- KMO were reconstituted in Soybean PC liposomes. Dimeric KMO was observed without the aid of cross-linking or additional reagents, suggesting that it is capable of tightly associating

itself to the liposome. (B) Cell-free assay data for liposome reconstituted KMO in PBS solution.

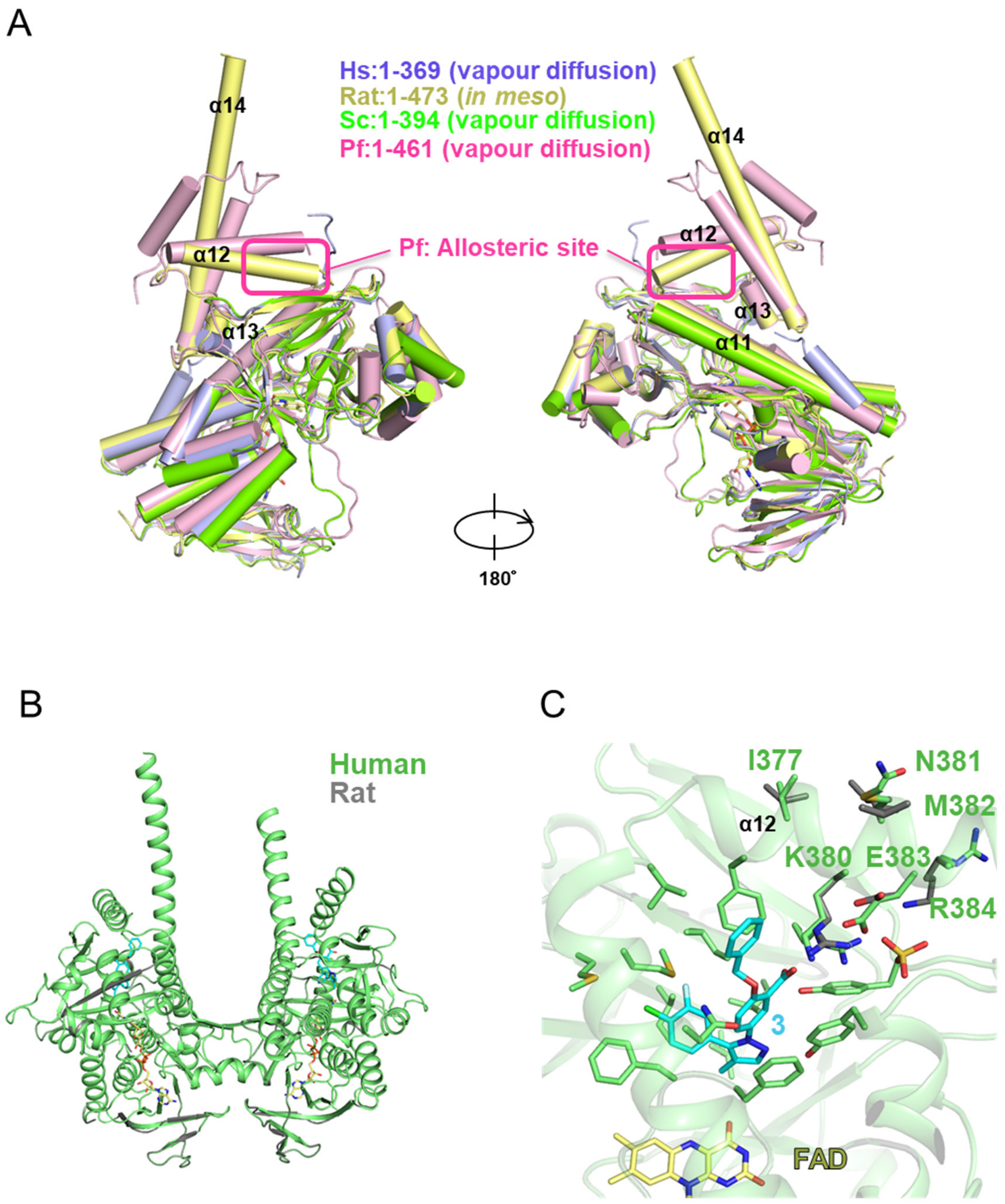

Supplementary Figure 6: Species differences between previously determined structures and Hs-KMO model structure.

(A) Superimposition of Rat-KMO determined in this study with previously determined structures. Rat, Human (Hs) (PDBID:5X68), *Saccharomyces cerevisiae* (Sc)

(PDBID:4J34) and *Pseudomonas fluorescens* (Pf) (PDBID:5MZC) KMO are coloured yellow, violet, green, and pink, respectively. The α12 and α14 helices underwent major structural rearrangements, and the allosteric pocket observed in Pf-KMO cannot be unambiguously identified. (B) Overall superimposition of the Hs-KMO model structure with that of Rat-KMO. Hs-KMO and Rat-KMO are shown in green and grey, respectively. (C) Superimposition of Hs-KMO and Rat-KMO in the catalytic pocket. All residues are conserved except for the five residues comprising the α12 helix.

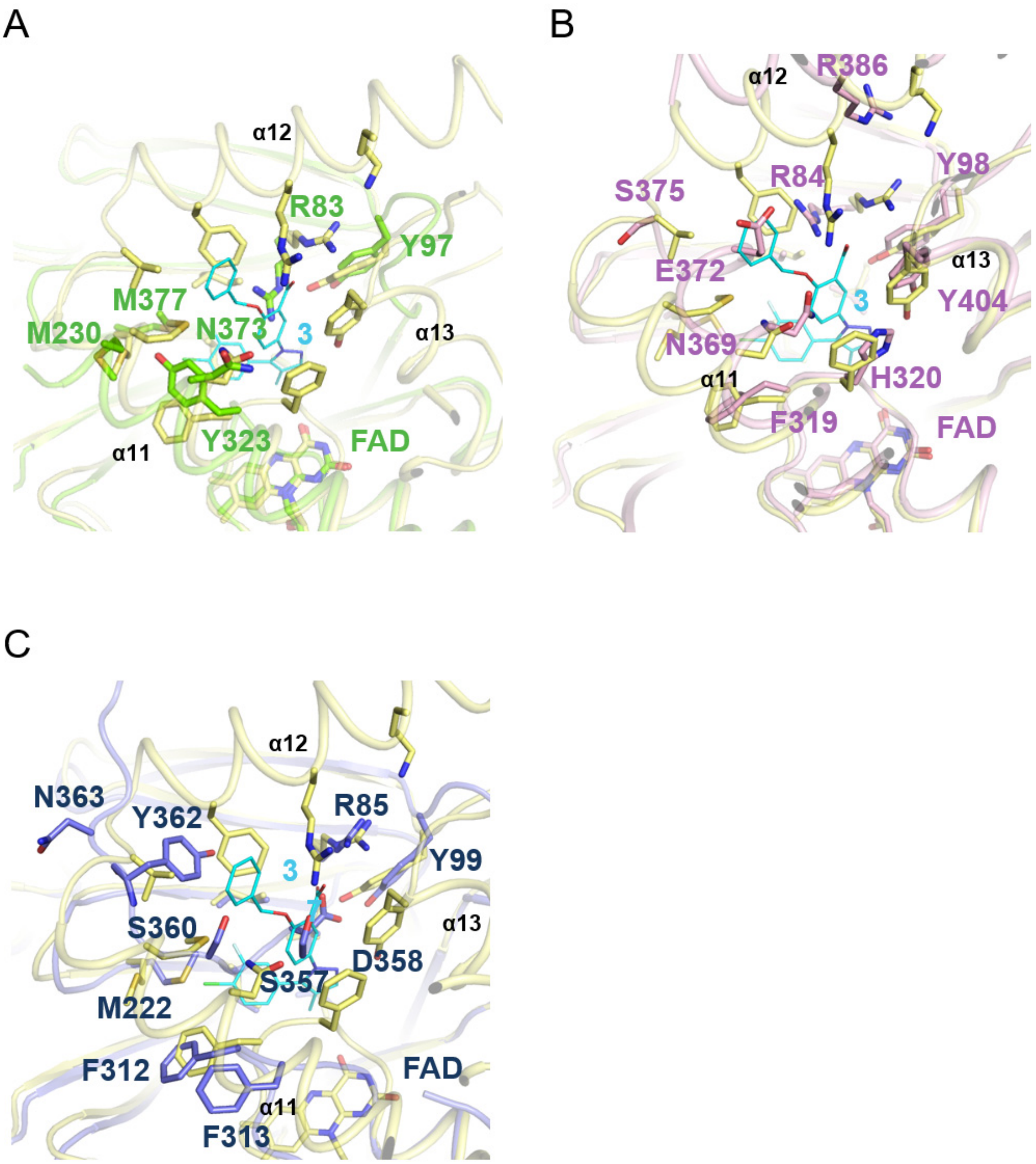

Supplementary Figure 7: Reactive cavity of Rat-KMO superimposed with previously determined KMO structures.

(A) Sc-KMO (PDBID:4J34), (B) Pf-KMO (PDBID:5MZC), and (C) Hs-KMO (PDBID:5X68) are shown in green, pink, and violet, respectively. Key residues are

depicted as sticks. Rat-KMO is shown as yellow cartoons and sticks. Compound **3** is shown as cyan sticks for reference.

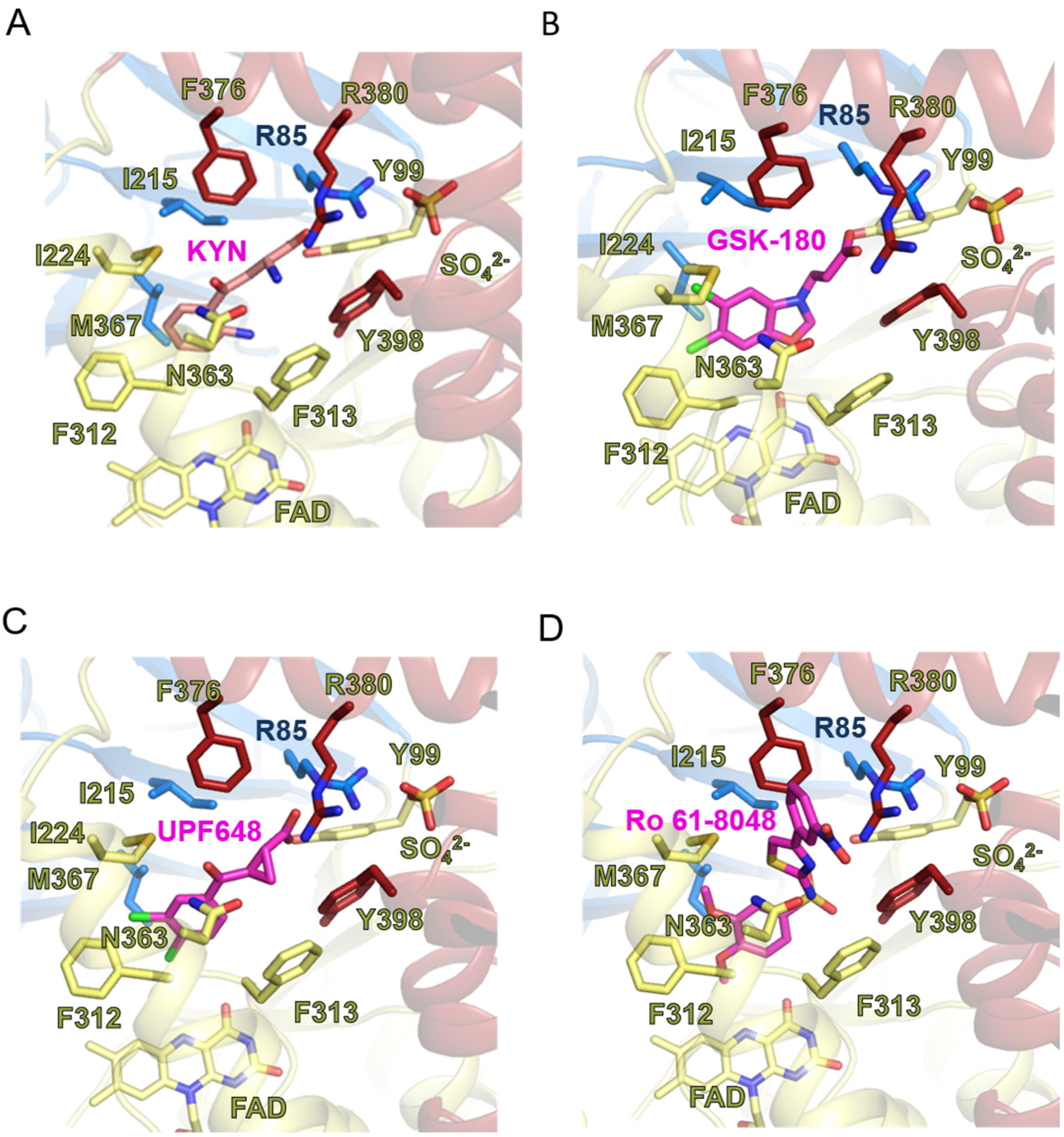

Supplementary Figure 8: Docking studies of KMO inhibitors.

Docking studies of (A) kynurenine (B) GSK-180, (C) UPF648, and (C) Ro-61-8048. The substrate kynurenine was used as a control to validate our docking study, which demonstrated interactions identified in previous reports. The centroid of compound **3**

was used as the docking site. Key residues and secondary structures are coloured using the same scheme as that in Figure 1. Key compounds are coloured magenta.

A

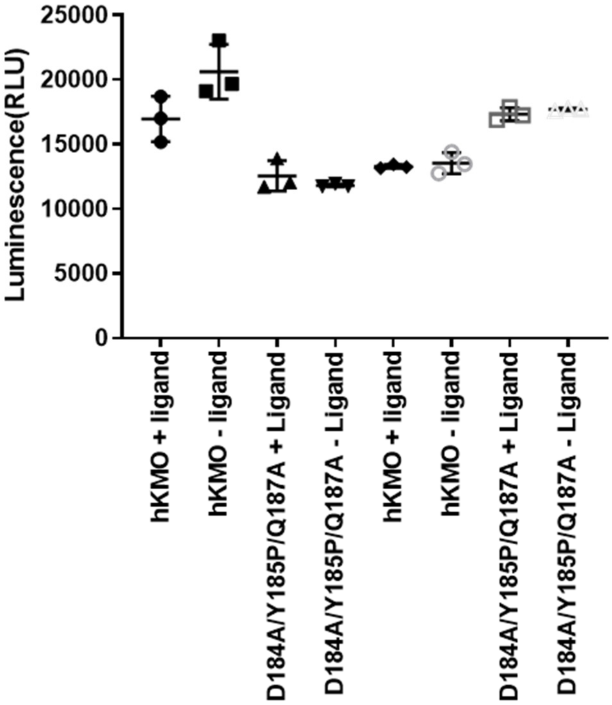

Luminescence(RLU)
25000
20000
15000
10000
5000
0
hKMO + ligand
hKMO - ligand
D184A/Y185P/Q187A + Ligand
D184A/Y185P/Q187A - Ligand
hKMO + ligand
hKMO - ligand
D184A/Y185P/Q187A + Ligand
D184A/Y185P/Q187A - Ligand

B

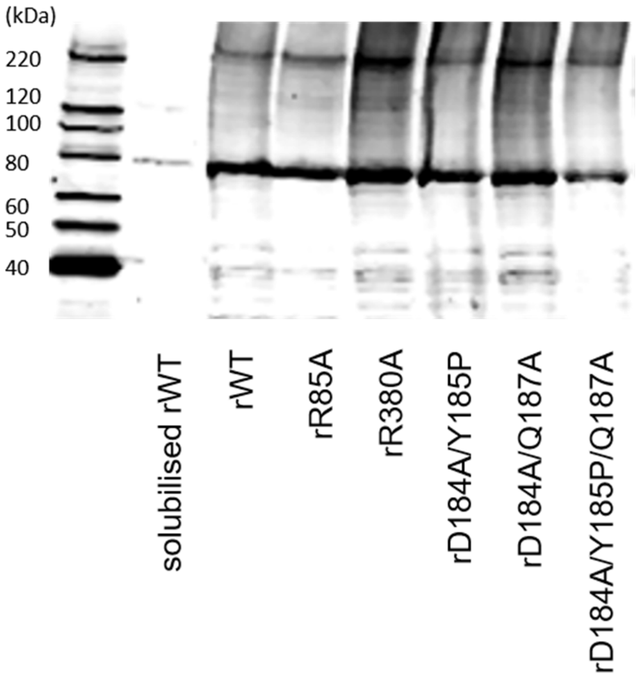

(kDa)
220
120
100
80
60
50
40
solubilised rWT
rWT
rR85A
rR380A
rD184A/Y185P
rD184A/Q187A
rD184A/Y185P/Q187A

Supplementary Figure 9:

(A) Viability of NanoBRET measured cells using Cell Titer-Glo 2.0. Mean triplicate luminescence values with and without the HaloTag NanoBRET 618 Ligand are shown. Error bars represent standard deviation.

(B) Quantification of KMO mitochondria membranes using anti-GST.

Reference	Ro 61-8048 IC_{50} (μM)	Kynurenine (μM)	Enzyme form	Detection method
This study	1.4	1200	recombinant protein	Absorbance
Jacobs *et al*	0.78	600	recombinant protein	Fluorescence
Winkler *et al*	0.035	20	liver extracts	LC/MS
Lowe *et al*	not shown	10	membrane extracts	LC/MS

Supplementary Table 1: Comparison of KMO inhibitor assay systems[1-3]. We performed assays using a higher concentration of kynurenine than that used in other reports to obtain highly potent compounds. Findings from our assay system are comparable to those reported by Jacobs *et al*, who demonstrated an IC_{50} value of 0.78 μM.

Reference	Ro 61-8048 IC_{50} (μM)	Kynurenine (μM)	Cells	Detection method
This study	1.2	100	HEK293	LC/MS
Winkler *et al*	0.64	100	CHO	LC/MS

Supplementary Table 2: Comparison of KMO cellular inhibitor assay systems. The results of our cellular assay are in good agreement with those of an independent report[2].

PDBID of all Ro 61-8048 complex structures	Species	Ro 61-8048 binding site
5X6Q	*Pseudomonas fluorescens*	Allosteric/Canonical
5Y66	*Pseudomonas fluorescens*	Allosteric
5X6R	*Saccharomyces cerevisiae*	Canonical

PDBID of Human KMO	Species	Inhibitor
5X68	*Homo sapiens*	None

Supplementary Table 3: The species and binding sites of reported Ro 61-8048-complexed KMO structures and human KMO structures[4,5].

Allosteric binding of Ro 61-8048 has only been confirmed for Pf-KMO. No inhibitor co-structures have been reported for the human KMO structure due to its autoinhibited nature.

	Signal intensity (RFU)
rWT	2.51E+3
rR85A	1.65E+3
rR380A	4.14E+3
rD184A/Y185P	1.90E+3
rD184A/Q187A	3.54E+3
rD184A/Y185P/Q187A	7.16E+2

Supplementary Table 4: Quantification of Rat-KMO mitochondrial membranes

Supplementary Note 1: Compound synthesis

^{1}H NMR and ^{13}C NMR spectra were recorded on Bruker AVANCE III HD500 or Varian VNS400, and chemical shifts are expressed in δ (ppm) values with trimethylsislane as an internal reference (s = singlet, d = doublet, t = triplet, r = quartet, m = multiplet, and br = broad peak). Mass spectra (MS) were recorded on a Waters UPLC/SQD LC/MS system. Electrospray ionization positive high resolution mass spectrometry (HRMS) was conducted using Thermo Scientific Exactive Plus. Column chromatography was performed on silica gel (Silica gel 60). Unless otherwise noted, all reagents and solvents obtained from commercial suppliers were used without further purification. The following abbreviations are used: CDI, carbonyldiimidazol; dba, dibenzylideneacetone; DBU, 1,8-diazabicyclo[5.4.0]undec-7-ene; DMF, *N*,*N*-dimethylformamide; DMSO, dimethylsulfoxide; dppf, 1,1'-bis(diphenylphosphino)ferrocene; EtOAc, ethyl acetate; EtOH, ethanol; IPE, diisopropyl ether; MeOH, methanol; SEM, 2-(trimethylsilyl)ethoxymethyl; TFA, trifluoroacetic acid; THF, tetrahydrofuran; XantPhos, 4,5-Bis(diphenylphosphino)-9,9-dimethylxanthene.

Preparation of **1**

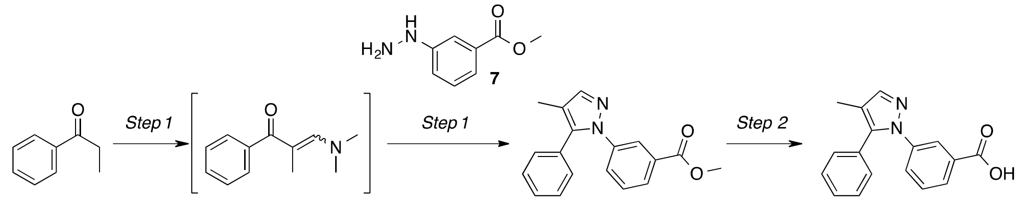

Step 1. **Methyl 3-(4-methyl-5-phenyl-1*H*-pyrazol-1-yl)benzoate (8)**

1,1-dimethoxy-*N*,*N*-dimethylmethanamine (1.0 mL, 7.50 mmol) was added to a mixture of 1-phenylpropan-1-one (**6**) (1.0 mL, 7.52 mmol) in DMF (15 mL) at room temperature. The mixture was stirred at 100 °C for 2 days. After cooling to room temperature, the reaction mixture was concentrated *in vacuo* to give the crude product (1.42 g, quantitative yield) as a yellow oil. The crude product was used for the next step without further purification.

AcOH (420 μL, 7.34 mmol) and methyl 3-hydrazinylbenzoate (**7**) (615mg, 3.70 mmol) were added to a mixture of the obtained crude product (700 mg, 3.70 mmol) in MeOH (12 mL). The mixture was stirred at 60 °C for 4 h. After cooling to room temperature, $CHCl_3$ and H_2O were added to the mixture. The mixture was extracted with $CHCl_3$. The combined organic layer was washed with brine, dried over Na_2SO_4, and concentrated *in vacuo.* The residue was purified by column chromatography on silica gel (*n*-hexane/EtOAc = 100/0 to 70/30) to give the product (220 mg, 20 %). ^{1}H NMR (DMSO-

d$_6$, 500 MHz): *δ* 2.06 (s, 3H), 3.82 (s, 3H), 7.19–7.24 (m, 2H), 7.34–7.48 (m, 5H), 7.70 (s, 1H), 7.79–7.86 (m, 2H); MS(ESI) *m/z* 293 $[M+H]^+$.

Step 2. **3-(4-Methyl-5-phenyl-1*H*-pyrazol-1-yl)benzoic acid (1)**

1 M NaOH aqueous solution (2.0 mL, 2.0 mmol) was added to a mixture of **8** (198 mg, 0.68 mmol) in MeOH (2.0 mL) and THF (2.0 mL). The mixture was stirred at room temperature for 3 h. After neutralising with 1 M HCl aqueous solution, $CHCl_3$ was added, and the mixture was extracted with $CHCl_3$. The combined organic layer was washed with brine, dried over Na_2SO_4, and concentrated *in vacuo*. The residue was purified by column chromatography on silica gel ($CHCl_3$/MeOH = 100/0 to 98/2) to give an amorphous solid. The amorphous solid was stirred in IPE/*n*-hexane = 1/1 at room temperature until a precipitate formed. The precipitate was collected and washed with IPE/*n*-hexane = 1/1 to give the product (134 mg, 71 %) as a colourless powder. ^{1}H NMR (DMSO-*d*$_6$, 400 MHz): *δ* 2.05 (s, 3H), 7.17–7.26 (m, 2H), 7.32–7.49 (m, 5H), 7.69 (s, 1H), 7.75–7.79 (m, 1H), 7.79–7.85 (m, 1H), 13.09 (br s, 1H); ^{13}C NMR (DMSO-*d*$_6$, 500 MHz): *δ* 9.31, 116.77, 125.32, 127.99, 128.77, 128.85, 129.21, 129.66, 130.05, 130.23, 132.09, 140.03, 140.52, 141.85, 166.87; MS(ESI) *m/z* 279 $[M+H]^+$; HRMS (ESI) *m/z* calcd for $C_{17}H_{15}N_2O_2$ ($[M+H]^+$): 279.1128, found: 279.1125.

<u>Preparation of **2**</u>

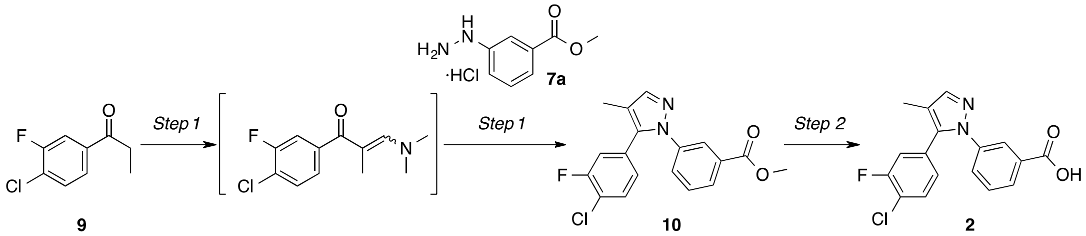

Step 1. **Methyl 3-[5-(4-chloro-3-fluorophenyl)-4-methyl-1*H*-pyrazol-1-yl]benzoate (10)**

A mixture of 1-(4-chloro-3-fluorophenyl)propan-1-one (**9**) (980 mg, 5.25 mmol) and 1,1-dimethoxy-*N*,*N*-dimethylmethanamine was stirred (3.5 mL, 26.0 mmol) at 100 °C for 2 days. The solvent was removed *in vacuo* to give the crude product (1.26 g, 99 %) as a brown oil. The crude product was used for the next step without further purification.

Methyl 3-hydrazinylbenzoate hydrogen chloride (**7a**) (300 mg, 1.48 mmol) was added to a solution of the crude product (375 mg, 1.55 mmol) in MeOH (5.0 mL). The mixture was stirred at 60 °C for 0.5 h. After cooling to room temperature, saturated $NaHCO_3$ aqueous solution was added, and the mixture was extracted with $CHCl_3$. The organic layer was dried over Na_2SO_4 and evaporated *in vacuo*. The crude mixture was purified twice by column chromatography on silica gel (*n*-hexane/EtOAc = 80/20 to 60/40) to give the product (340 mg, 67 %) as a pale yellow oil. ^{1}H NMR (DMSO-d_6, 400 MHz): δ 2.07 (s,

3H), 3.84 (s, 3H), 7.03–7.08 (m, 1H), 7.35–7.43 (m, 2H), 7.51 (t, J = 7.9 Hz, 1H), 7.63 (t, J = 8.1 Hz, 1H), 7.72 (s, 1H), 7.83–7.91 (m, 2H); MS(ESI) *m/z* 345 $[M+H]^+$.

Step 2. **3-[5-(4-Chloro-3-fluorophenyl)-4-methyl-1H-pyrazol-1-yl]benzoic acid (2)**

1 M NaOH aqueous solution (1.3 mL, 1.3 mmol) was added to a mixture of **10** (144 mg, 0.42 mmol) in MeOH (2.0 mL) and THF (2.0 mL) at room temperature. The mixture was stirred at room temperature for 2 h. After neutralising with 1 M HCl aqueous solution, H_2O and $CHCl_3$ were added, and the mixture was extracted with $CHCl_3$. The organic layer was washed with brine, dried over Na_2SO_4 and evaporated *in vacuo*. The crude mixture was purified by column chromatography on silica gel ($CHCl_3$/MeOH = 100/0 to 95/5) to give an amorphous solid. The amorphous solid was stirred in IPE/*n*-hexane = 1/1 at room temperature until a precipitate formed. The precipitate was collected and washed with IPE/*n*-hexane = 1/1 to give the product (35 mg, 25 %) as a colourless powder. ^{1}H NMR (DMSO-d_6, 500 MHz): δ 2.07 (s, 3H), 7.02–7.07 (m, 1H), 7.36–7.41 (m, 2H), 7.49 (t, J = 7.9 Hz, 1H), 7.62 (t, J = 8.1 Hz, 1H), 7.72 (s, 1H), 7.78–7.82(m, 1H), 7.84–7.88 (m, 1H), 13.16 (br s, 1H); ^{13}C NMR (DMSO-d_6, 500 MHz): δ .9.21, 117.60, 118.41, 118.58, 120.08, 120.22, 125.45, 127.58, 127.60, 128.30, 128.93, 129.87, 131.23, 131.29, 131.48, 132.22, 137.83, 137.84, 140.10, 141.97, 156.53, 158.49, 166.84; MS(ESI) *m/z* 331, 333

$[M+H]^+$; HRMS (ESI) *m/z* calcd for $C_{17}H_{13}N_2O_2FCl$ ($[M+H]^+$): 331.0644, found: 331.0644.

Preparation of **3**

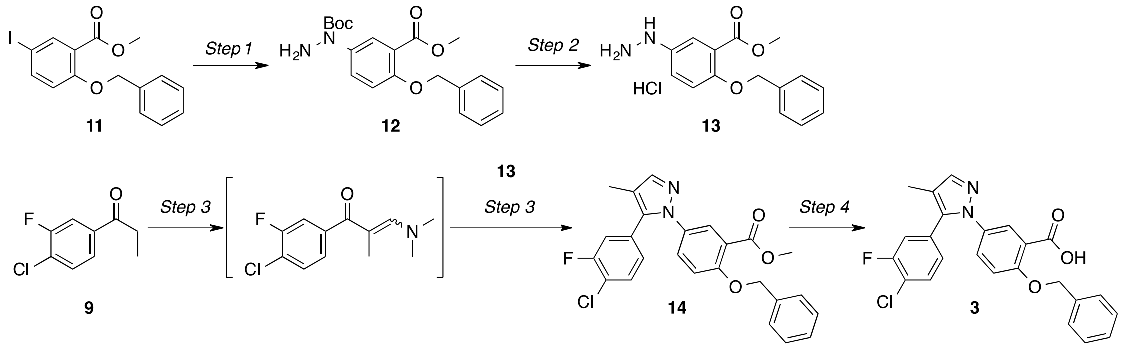

Step 1. ***tert*-butyl 1-[4-(benzyloxy)-3-(methoxycarbonyl)phenyl]hydrazine-1-carboxylate (12)**

A mixture of methyl 2-(benzyloxy)-5-iodobenzoate (**11**) (1.32 g, 3.59 mmol), *tert*-butyl hydrazinecarboxylate (616 mg, 4.66 mmol), CuI (68 mg, 0.36 mmol) and Cs_2CO_3 (1.75 g, 5.37 mmol) in DMSO (7.2 mL) was stirred at 50 °C for 3 h under an Ar atmosphere. After cooling to room temperature, H_2O and saturated NH_4Cl aqueous solution were added to the reaction mixture. The mixture was extracted with EtOAc. The organic layer was washed with brine, dried over Na_2SO_4 and concentrated *in vacuo*. The residue was purified by column chromatography on silica gel (*n*-hexane/AcOEt = 80/20 to 50/50) to give the product (1.31 g, 98 %) as a colourless oil. ^{1}H NMR (DMSO-d_6, 500 MHz): δ 1.44 (s, 9H), 3.80 (s, 3H), 5.06 (s, 2H), 5.18 (s, 2H), 7.15 (d, J = 9.1 Hz, 1H), 7.29–7.34

(m, 1H), 7.37–7.42 (m, 2H), 7.46–7.52 (m, 2H), 7.54–7.60 (m, 1H), 7.79 (d, *J* = 2.8 Hz, 1H); MS (ESI) *m/z* 395 $[M+Na]^+$.

Step 2. **Methyl 2-(benzyloxy)-5-hydrazinylbenzoate hydrogen chloride (13)**

A solution of 4 M HCl in 1,4-dioxane (10 mL) was added to a mixture of **12** (1.31 g, 3.52 mmol) in MeOH (10 mL). The mixture was stirred at room temperature for 4 h and then at 50 °C for 3 h. After cooling to temperature, the mixture was concentrated *in vacuo* to give the product (990 mg, 91 %) as a yellow powder. ^{1}H NMR (DMSO-d_6, 500 MHz): δ 3.82 (s, 3H), 5.16 (s, 2H), 7.17–7.23 (m, 2H), 7.28–7.33 (m, 1H), 7.35–7.42 (m, 3H), 7.43–7.50 (m, 2H), 7.93–8.26 (m, 1H), 9.95–10.24 (m, 3H); MS(ESI) *m/z* 273 $[M+H\text{-}HCl]^+$.

Step 3. **Methyl 2-(benzyloxy)-5-[5-(4-chloro-3-fluorophenyl)-4-methyl-1*H*-pyrazol-1-yl]benzoate (14)**

A mixture of **9** (5.0 g, 27 mmol) and 1,1-dimethoxy-*N*,*N*-dimethylmethanamine (18 mL, 135 mmol) was stirred at 100 °C for 3 days. The solvent was removed *in vacuo* to give the crude product (6.5 g, quantitative yield) as a brown oil. The crude product was used for the next step without further purification.

A mixture of the crude product (4.31 g, 17.8 mmol), MeOH (40 mL), and **13** (5.00 g, 16.2 mmol) was stirred at 50 °C for 0.5 h. After cooling to room temperature, saturated $NaHCO_3$ aqueous solution was added to the reaction mixture. The mixture was extracted with $CHCl_3$. The organic layer was dried over Na_2SO_4 and evaporated *in vacuo*. The crude mixture was purified by column chromatography on silica gel (*n*-hexane/EtOAc = 80/20 to 70/30) to give the product (5.00 g, 68 %) as an orange oil. ^{1}H NMR (DMSO-d_6, 500 MHz): δ 2.06 (s, 3H), 3.78 (s, 3H), 5.21 (s, 2H), 7.01–7.06 (m, 1H), 7.18–7.23 (m, 1H), 7.24–7.28 (m, 1H), 7.29–7.42 (m, 4H), 7.44–7.49 (m, 2H), 7.59–764 (m, 2H), 7.66 (s, 1H); MS(ESI) *m/z* 451 $[M+H]^+$.

Step 4. **2-(benzyloxy)-5-[5-(4-chloro-3-fluorophenyl)-4-methyl-1*H*-pyrazol-1-yl]benzoic acid (3)**

1 M NaOH aqueous solution (4.0 mL, 4.0 mmol) was added to a mixture of **14** (300 mg, 0.67 mmol) in MeOH (4.0 mL). The mixture was stirred at 50 °C for 0.5 h. A 1 M HCl aqueous solution was added, and the mixture was extracted with $CHCl_3$. The organic layer was dried over Na_2SO_4 and evaporated *in vacuo*. The crude mixture was purified by column chromatography on silica gel ($CHCl_3$/MeOH = 100/0 to 95/5). The obtained solid was washed with IPE to give the product (270 mg, 93 %) as a white solid. ^{1}H NMR (DMSO-d_6, 400 MHz): δ 2.06 (s, 3H), 5.19 (s, 2H), 7.01–7.07 (m, 1H), 7.15–7.21 (m,

1H), 7.22–7.27 (m, 1H), 7.28–7.42 (m, 4H), 7.44–7.50 (m, 2H), 7.55 (d, J = 2.8 Hz, 1H), 7.58–7.67 (m, 2H), 12.83 (br s, 1H); ^{13}C NMR (DMSO-d_6, 500 MHz): δ 9.27, 70.38, 114.71, 116.92, 118.41, 118.58, 119.92, 120.06, 122.47, 127.49, 127.57, 127.60, 127.65, 128.20, 128.82, 129.41, 131.32, 131.39, 132.73, 137.16, 137.82, 137.83, 141.37, 156.41, 156.47, 158.44, 166.78; MS(ESI) *m/z* 437 $[M+H]^+$; HRMS (ESI) *m/z* calcd for $C_{24}H_{19}N_2O_3FCl$ ($[M+H]^+$): 437.1063, found: 437.1065.

Preparation of **4**

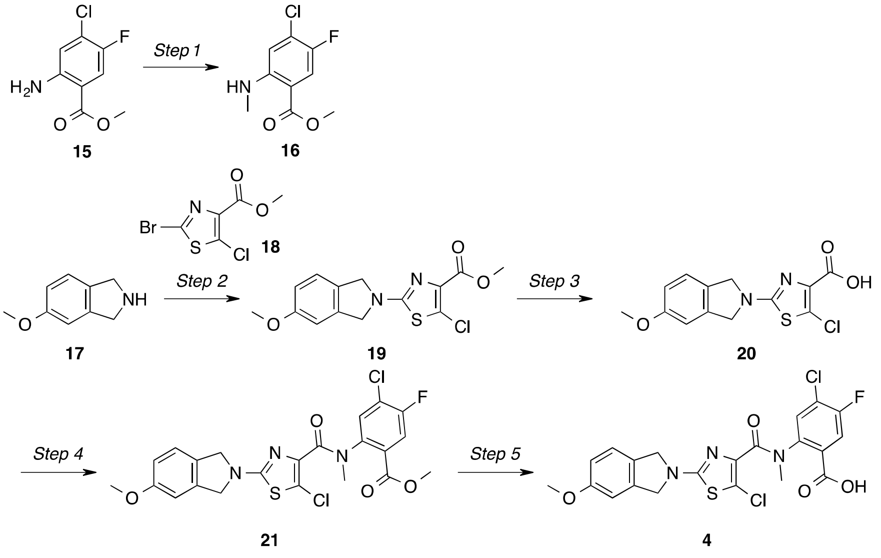

Step 1. **Methyl 4-chloro-5-fluoro-2-(methylamino)benzoate (16)**

NaOH (354 mg, 8.85 mmol) and MeI (0.51 mL, 8.20 mmol) were added to a mixture of methyl 2-amino-4-chloro-5-fluorobenzoate (**15**) (1.50 g, 7.37 mmol) in DMF (9.0 mL). The mixture was stirred at 50 °C for 24 h. H_2O was added, and the mixture was extracted with EtOAc. The organic layer was washed with brine, dried over Na_2SO_4 and evaporated *in vacuo*. The crude mixture was purified by column chromatography on silica gel (*n*-hexane/EtOAc = 90/10 to 85/15) to give the product (725 mg, 45 %) as a yellow solid. ^{1}H NMR (DMSO-d_6, 500 MHz): δ 2.84 (d, J = 5.1 Hz, 3H), 3.81 (s, 3H), 6.85 (d, J = 6.4 Hz, 1H), 7.43–7.52 (m, 1H), 7.66 (d, J = 10.2 Hz, 1H); MS(ESI) *m/z*: 218 [M+H]$^+$.

Step 2. **Methyl 5-chloro-2-(5-methoxy-1,3-dihydro-2*H*-isoindol-2-yl)-1,3-thiazole-4-carboxylate (19)**

A mixture of 5-methoxy-2,3-dihydro-1*H*-isoindole (**17**) (500 mg, 3.35 mmol), methyl 2-bromo-5-chloro-1,3-thiazole-4-carboxylate (**18**) (903 mg, 3.52 mmol), $Pd_2(dba)_3$ (307 mg, 0.34 mmol), XantPhos (194 mg, 0.34 mmol), K_2CO_3 (464 mg, 3.36 mmol) and DMF (10 mL) was stirred at 100 °C for 3 h. The mixture was concentrated *in vacuo*. The crude mixture was purified by column chromatography on silica gel (*n*-hexane/EtOAc = 70/30 to 50/50). The obtained powder was washed with IPE to give the product (550 mg, 51 %) as a pale yellow powder. ^{1}H NMR (DMSO-d_6, 500 MHz): δ 3.76 (s, 3H), 3.81 (s, 3H), 4.65 (s, 2H), 4.70 (s, 2H), 6.88–6.93 (m, 1H), 6.97–7.00 (m, 1H), 7.30 (d, J = 8.4 Hz, 1H); MS(ESI) *m/z*: 325 $[M+H]^+$.

Step 3. **5-chloro-2-(5-methoxy-1,3-dihydro-2*H*-isoindol-2-yl)-1,3-thiazole-4-carboxylic acid (20)**

1 M NaOH aqueous solution (5.0 mL, 5.0 mmol) was added to a mixture of **19** (550 mg, 1.69 mmol) in MeOH (5.0 mL) and THF (5.0 mL). The mixture was stirred at room temperature for 3 h. A 1 M HCl aqueous solution was added to the mixture. The solids were collected by filtration. The obtained solid was washed with H_2O to give the product

(526 mg, quantitative yield) as a white powder. ^{1}H NMR (DMSO-d_6, 500 MHz): δ 3.77 (s, 3H), 4.64 (s, 2H), 4.69 (s, 2H), 6.91 (dd, J = 2.5, 8.4 Hz, 1H), 6.97–7.01 (m, 1H), 7.30 (d, J = 8.4 Hz, 1H), 13.09 (br s, 1H); MS(ESI) *m/z*: 311 [M+H]$^+$.

Step 4. **Methyl 4-chloro-2-{[5-chloro-2-(5-methoxy-1,3-dihydro-2*H*-isoindol-2-yl)-1,3-thiazole-4-carbonyl](methyl)amino}-5-fluorobenzoate (21)**

$POCl_3$ (88 µL, 0.96 mmol) was added to a mixture of **20** (300 mg, 0.97 mmol), **16** (210 mg, 0.96 mmol) and pyridine (10 mL). The mixture was stirred at room temperature for 5 h. The reaction mixture was concentrated *in vacuo*. The crude mixture was purified by column chromatography on silica gel ($CHCl_3$/MeOH = 99/1 to 95/5). The obtained powder was washed with 2-propanol to give the product (190 mg, 39 %) as a white powder. ^{1}H NMR (DMSO-d_6, 500 MHz): δ 3.32 (s, 3H), 3.75–3.77 (m, 3H), 3.83–3.85 (m, 3H), 4.27–4.51 (m, 4H), 6.87–6.95 (m, 2H), 7.24 (d, J = 8.4 Hz, 1H), 7.67–7.74 (m, 1H), 7.92–7.97 (m, 1H); MS(ESI) *m/z*: 510 [M+H]$^+$.

Step 5. **4-Chloro-2-{[5-chloro-2-(5-methoxy-1,3-dihydro-2*H*-isoindol-2-yl)-1,3-thiazole-4-carbonyl](methyl)amino}-5-fluorobenzoic acid (4)**

1 M NaOH aqueous solution (1.2 mL, 1.2 mmol) was added to a mixture of **21** (190 mg, 0.37 mmol), MeOH (2.0 mL) and THF (2.0 mL). The solution was stirred at 50 °C

for 3 h. After cooling to room temperature, 1 M HCl aqueous solution was added to the mixture. The solid was collected by filtration. The obtained solids were washed with EtOH to give the product (170 mg, 92 %) as a white powder. ^{1}H NMR (DMSO-d_6, 500 MHz): δ 3.28 (s, 3H), 3.76 (s, 3H), 4.25–4.51 (m, 4H), 6.86–6.94 (m, 2H), 7.19–7.25 (m, 1H), 7.69 (d, J = 9.5 Hz, 1H), 7.86 (d, J = 6.7 Hz, 1H), 13.46 (br s, 1H); ^{13}C NMR (DMSO-d_6, 500 MHz): δ 36.31, 53.31, 53.91, 54.75, 107.33, 111.67, 113.40, 117.38, 117.56, 122.29, 122.44, 122.97, 127.08, 129.62, 129.68, 132.06, 136.82, 139.49, 139.52, 141.34, 154.12, 156.10, 158.64, 160.59, 160.82, 163.89, 163.90; MS(ESI) *m/z* 496 $[M+H]^+$; HRMS (ESI) *m/z* calcd for $C_{21}H_{17}N_3O_4SFCl_2$ ($[M+H]^+$): 496.0295, found: 496.0299.

Preparation of **5**

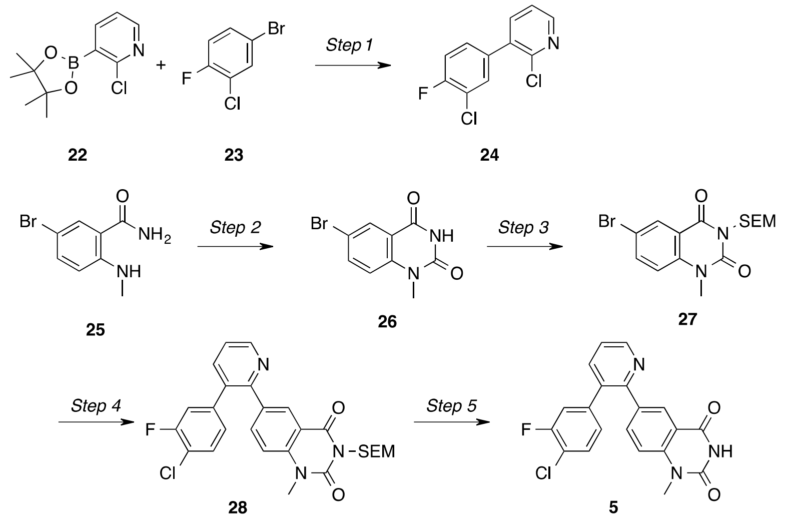

Step 1. **2-chloro-3-(4-chloro-3-fluorophenyl)pyridine (24)**

A mixture of 2-chloro-3-(4,4,5,5-tetramethyl-1,3,2-dioxaborolan-2-yl)pyridine (**22**) (300 mg, 1.25 mmol), 4-bromo-1-chloro-2-fluorobenzene (**23**) (0.17 mL, 1.40 mmol), Na_2CO_3 (265 mg, 2.50 mmol), 1,4-dioxane (3.0 mL), H_2O (0.6 mL), and $Pd(PPh_3)_4$ (73 mg, 0.063 mmol) was stirred under an Ar atmosphere at 90 °C for 1 h. After cooling to room temperature, H_2O was added, and the mixture was extracted with EtOAc. The organic layer was dried over Na_2SO_4 and evaporated *in vacuo*. The crude mixture was purified by column chromatography on silica gel (*n*-hexane/EtOAc = 80/20 to 70/30) to give the product (240 mg, 79 %) as a pale yellow solid. ^1H NMR (DMSO-d_6, 500 MHz): δ 7.35–7.40 (m, 1H), 7.56 (dd, J = 4.9, 7.7 Hz, 1H), 7.63 (dd, J = 2.1, 10.3Hz, 1H), 7.72

(t, J = 8.0 Hz, 1H), 7.94 (dd, J = 2.0, 7.5 Hz, 1H), 8.48 (dd, J = 2.0, 4.7 Hz, 1H); MS(ESI) *m/z*: 242, 244 $[M+H]^+$.

Step 2. **6-bromo-1-methylquinazoline-2,4(1*H*,3*H*)-dione (26)**

DBU (1.0 mL, 6.69 mmol) and CDI (1.0 g, 6.17 mmol) were added to a mixture of 5-bromo-2-(methylamino)benzamide (**25**) (940 mg, 4.10 mmol) in THF (15 mL). The mixture was stirred at room temperature overnight. H_2O and 1 M HCl aqueous solution were added to the mixture. The solids were collected by filtration to give the product (910 mg, 87 %) as a pale yellow powder. ^1H NMR (DMSO-d_6, 400 MHz): δ 3.42 (s, 3H), 7.40 (d, J = 8.9 Hz, 1H), 7.91 (dd, J = 2.5, 8.9 Hz, 1H), 8.04 (d, J = 2.4 Hz, 1H), 11.69 (br s, 1H); MS(ESI) *m/z*: 255, 257 $[M+H]^+$.

Step 3. **6-bromo-1-methyl-3-{[2-(trimethylsilyl)ethoxy]methyl}quinazoline-2,4(1*H*,3*H*)-dione (27)**

NaH (55 % dispersion in mineral oil, 200 mg, 4.58 mmol) was added to a mixture of **26** (910 mg, 3.57 mmol) in DMF (12 mL) under a nitrogen atmosphere in an ice bath. The mixture was stirred at the same temperature for 30 min. SEMCl (760 μL, 4.31 mmol) was added, and the mixture was stirred at room temperature for 4 h. H_2O and EtOAc were added, and the mixture was extracted with EtOAc. The organic layer was washed with

brine, dried over Na_2SO_4 and evaporated *in vacuo*. *n*-hexane/EtOAc =2/1 was added to the obtained solid and the mixture was stirred in an ice bath. The solids were collected by filtration to give the product (978 mg, 71 %) as a colourless solid. ^{1}H NMR (DMSO-d_6, 400 MHz): δ –0.04 (s, 9H), 0.86 (t, J = 8.1 Hz, 2H), 3.50 (s, 3H), 3.62 (t, J = 8.1 Hz, 2H), 5.35 (s, 2H), 7.44 (d, J = 8.9 Hz, 1H), 7.95 (dd, J = 2.4, 9.0 Hz, 1H), 8.12 (d, J = 2.4 Hz, 1H); MS(ESI) *m/z*: 407, 409 $[M+Na]^+$.

Step 4. **6-[3-(4-chloro-3-fluorophenyl)pyridin-2-yl]-1-methyl-3-{[2-(trimethylsilyl)ethoxy]methyl}quinazoline-2,4(1*H*,3*H*)-dione (28)**

A mixture of **27** (340 mg, 0.88 mmol), bis(pinacolato)diboron (670 mg, 2.64 mmol), $PdCl_2$(dppf)·CH_2Cl_2 (70 mg, 0.0086 mmol), KOAc (260 mg, 2.65 mmol) and 1,4-dioxane (7.0 mL) was stirred at 80 °C for 4 h under an N_2 atmosphere. After cooling to room temperature, **24** (370 mg, 1.52 mmol), $PdCl_2$(dppf)·CH_2Cl_2 (50 mg, 0.0061 mmol), Cs_2CO_3 (870 mg, 2.67 mmol) and H_2O (2.0 mL) were added, and the mixture was stirred at 100 °C for 1.5 h under an N_2 atmosphere. After cooling to room temperature, the mixture was diluted with EtOAc. The mixture was filtered through a Celite pad. EtOAc and H_2O were added to the filtrate, and the mixture was extracted with EtOAc. The organic layer was washed with brine, dried over Na_2SO_4 and evaporated *in vacuo*. The crude mixture was purified by column chromatography on silica gel (*n*-hexane/EtOAc =

80/20 to 0/100) to give the product (280 mg, 62 %) as a pale brown oil. ^{1}H NMR (DMSO-d_6, 500 MHz): δ –0.05 (s, 9H), 0.81–0.89 (m, 2H), 3.50 (s, 3H), 3.55–3.65 (m, 2H), 5.34 (s, 2H), 7.04–7.10 (m, 1H), 7.36–7.46 (m, 2H), 7.49–7.58 (m, 2H), 7.69 (dd, J = 2.3, 8.8 Hz, 1H), 7.89 (dd, J = 1.6, 7.8 Hz, 1H), 8.09 (d, J = 2.1 Hz, 1H), 8.74 (dd, J = 1.7, 4.7 Hz, 1H); MS(ESI) *m/z*: 512 $[M+H]^+$.

Step 5. **6-[3-(4-chloro-3-fluorophenyl)pyridin-2-yl]-1-methylquinazoline-2,4(1*H*,3*H*)-dione (5)**

TFA (4.0 mL) was added to a mixture of **28** (405 mg, 0.79 mmol) in CH_2Cl_2 (4.0 mL). The mixture was stirred at room temperature overnight. The mixture was concentrated *in vacuo*. $CHCl_3$ (4.0 mL) and MeOH (4.0 mL) were added to the residue. Ethylenediamine (530 μL, 7.91 mmol) was added, and the mixture was stirred at room temperature for 1 h. $CHCl_3$ and 1 M HCl aqueous solution were added, and the mixture was extracted with $CHCl_3$. The organic layer was dried over Na_2SO_4 and evaporated *in vacuo*. The crude mixture was purified by column chromatography on silica gel ($CHCl_3$/MeOH = 100/0 to 90/10) to give the product (217 mg, 72 %) as a colourless amorphous solid. ^{1}H NMR (DMSO-d_6, 400 MHz): δ 3.42 (s, 3H), 7.05 (dd, J = 1.8, 8.2 Hz, 1H), 7.34 (d, J = 8.8 Hz, 1H), 7.42 (dd, J = 2.0, 10.4 Hz, 1H), 7.48–7.58 (m, 2H), 7.61 (dd, J = 2.2, 8.7 Hz, 1H), 7.89 (dd, J = 1.7, 7.8 Hz, 1H), 8.73 (dd, J = 1.6, 4.9 Hz, 1H), 8.06 (d, J = 2.3 Hz, 1H),

11.55 (br s, 1H); ^{13}C NMR (DMSO-d_6, 500 MHz): δ 28.99, 113.80, 114.72, 117.16, 117.33, 118.08, 118.22, 122.07, 126.28, 126.31, 128.09, 130.13, 132.62, 132.63, 132.91, 135.61, 138.31, 139.97, 140.03, 140.74, 148.55, 149.58, 153.68, 155.50, 157.46, 160.91; MS(ESI) *m/z*: 382 $[M+H]^+$; HRMS (ESI) *m/z* calcd for $C_{20}H_{14}N_3O_2FCl$ ($[M+H]^+$): 382.0753, found: 382.0754.

Uncropped image of Supplementary Figure 2b.

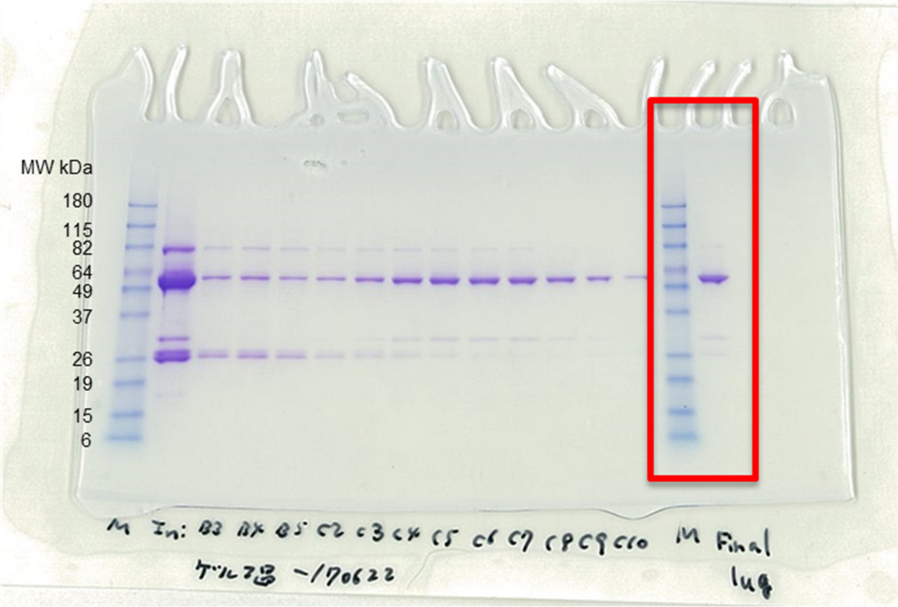

Uncropped image of Supplementary Figure 5a

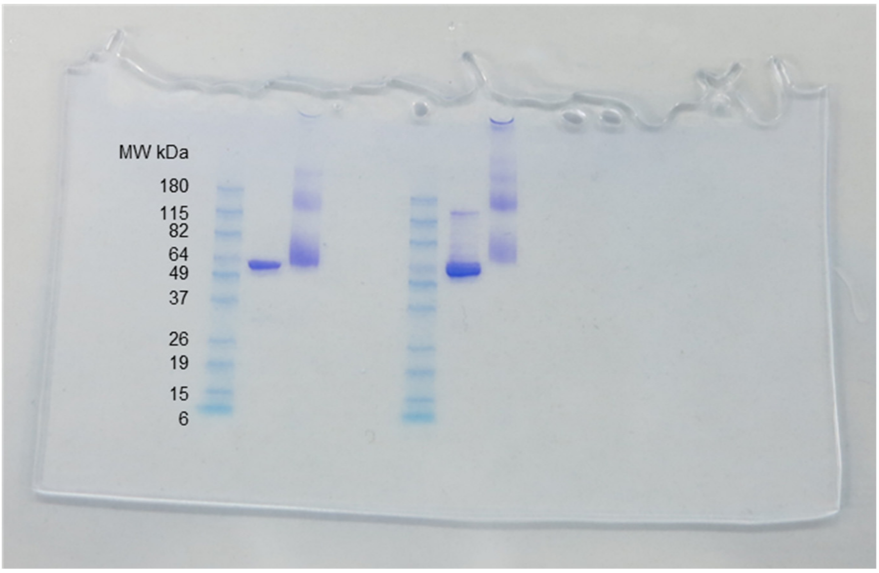

Uncropped image of Supplementary Figure 9b.

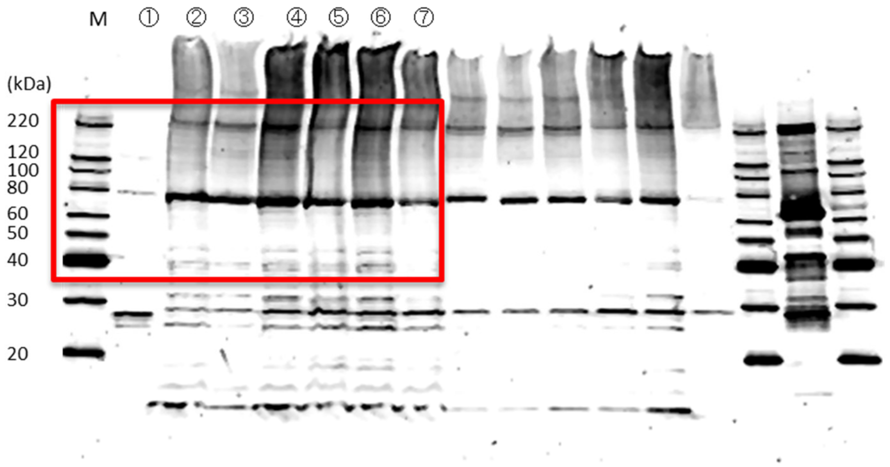

M:Marker*
Lane 1:solubilized rWT
Lane 2:rWT
Lane 3:rR85A
Lane 4:rR380A
Lane 5:rD184A/Y185P
Lane 6:rD184A/Q187A
Lane 7:rD184A/Y185P/Q187A

*MagicMark™ XP Western Protein Standard (Invitrogen Cat.LC5602)
Novex® Sharp Pre-Stained Protein Standard(Invitrogen Cat.LC5800)
was mixed at a 1:2 ratio.

References

1 Jacobs, K. R., Guillemin, G. J. & Lovejoy, D. B. Development of a Rapid Fluorescence-Based High-Throughput Screening Assay to Identify Novel Kynurenine 3-Monooxygenase Inhibitor Scaffolds. *SLAS Discov* **23**, 554-560, doi:10.1177/2472555218757180 (2018).

2 Winkler, D. *et al.* Development of LC/MS/MS, high-throughput enzymatic and cellular assays for the characterization of compounds that inhibit kynurenine monooxygenase (KMO). *Journal of biomolecular screening* **18**, 879-889, doi:10.1177/1087057113489731 (2013).

3 Lowe, D. M. *et al.* Lead discovery for human kynurenine 3-monooxygenase by high-throughput RapidFire mass spectrometry. *Journal of biomolecular screening* **19**, 508-515, doi:10.1177/1087057113518069 (2014).

4 Kim, H. T. *et al.* Structural Basis for Inhibitor-Induced Hydrogen Peroxide Production by Kynurenine 3-Monooxygenase. *Cell chemical biology* **25**, 426-438 e424, doi:10.1016/j.chembiol.2018.01.008 (2018).

5 Gao, J. *et al.* Biochemistry and structural studies of kynurenine 3-monooxygenase reveal allosteric inhibition by Ro 61-8048. *FASEB journal : official publication of the Federation of American Societies for Experimental Biology* **32**, 2036-2045, doi:10.1096/fj.201700397RR (2018).
