# Supplementary material for: Full-length in meso structure and mechanism of rat kynurenine 3-monooxygenase inhibition
Source: Commun Biol. 2021 Feb 4;4:159. doi: 10.1038/s42003-021-01666-5 (PMC7862291; doi:10.1038/s42003-021-01666-5)
Supplement: Supplementary file 2 — Description of Additional Supplementary Files [file 42003_2021_1666_MOESM2_ESM.pdf]

Description of Additional Supplementary Files

File Name: Supplementary Data 1
Description: Source data for Supplementary Figure 2a.

File Name: Supplementary Data 2
Description: Source data for all other graphs.
