# Supplementary material for: Full-length in meso structure and mechanism of rat kynurenine 3-monooxygenase inhibition
Source: Commun Biol. 2021 Feb 4;4:159. doi: 10.1038/s42003-021-01666-5 (PMC7862291; doi:10.1038/s42003-021-01666-5)
Supplement: Supplementary file 5 — Reporting Summary [file 42003_2021_1666_MOESM5_ESM.pdf]

nature research

Corresponding author(s): Shinya Mimasu

Last updated by author(s): Nov 17, 2020

Reporting Summary

Nature Research wishes to improve the reproducibility of the work that we publish. This form provides structure for consistency and transparency in reporting. For further information on Nature Research policies, see Authors & Referees and the Editorial Policy Checklist .

Statistics

For all statistical analyses, confirm that the following items are present in the figure legend, table legend, main text, or Methods section.

n/a	Confirmed	
☐	☒	The exact sample size (*n*) for each experimental group/condition, given as a discrete number and unit of measurement
☐	☒	A statement on whether measurements were taken from distinct samples or whether the same sample was measured repeatedly
☐	☒	The statistical test(s) used AND whether they are one- or two-sided<br>*Only common tests should be described solely by name; describe more complex techniques in the Methods section.*
☒	☐	A description of all covariates tested
☒	☐	A description of any assumptions or corrections, such as tests of normality and adjustment for multiple comparisons
☐	☒	A full description of the statistical parameters including central tendency (e.g. means) or other basic estimates (e.g. regression coefficient) AND variation (e.g. standard deviation) or associated estimates of uncertainty (e.g. confidence intervals)
☐	☒	For null hypothesis testing, the test statistic (e.g. *F*, *t*, *r*) with confidence intervals, effect sizes, degrees of freedom and *P* value noted<br>*Give P values as exact values whenever suitable.*
☒	☐	For Bayesian analysis, information on the choice of priors and Markov chain Monte Carlo settings
☒	☐	For hierarchical and complex designs, identification of the appropriate level for tests and full reporting of outcomes
☒	☐	Estimates of effect sizes (e.g. Cohen's *d*, Pearson's *r*), indicating how they were calculated

Our web collection on statistics for biologists contains articles on many of the points above.

Software and code

Policy information about availability of computer code

Data collection	X-ray data was collected at SPring-8 BL-32XU using KUMA and SHIKA.
Data analysis	X-ray data was analyzed using:<br>KAMO(XDS, BLEND)<br>CCP4<br>Pymol was used to generate figure images of determined structures.<br>Coot was used for modelling.<br>Molecular Operating Environment (MOE) was used to generate ligand-protein interaction figures.<br>AFITT (Openeye) was used for ligand fitting and to generate cif files.<br>Schrodinger Maestro was used for homology modelling and molecular docking.<br>Assays and statistical analysis were analyzed using GraphPad Prism for IC50 values.<br>Western blots were analyzed in ImageStudio2.0 (LI-COR Biosciences).<br>All references are given in the Material and Methods section

For manuscripts utilizing custom algorithms or software that are central to the research but not yet described in published literature, software must be made available to editors/reviewers. We strongly encourage code deposition in a community repository (e.g. GitHub). See the Nature Research guidelines for submitting code & software for further information.

Data

Policy information about availability of data

All manuscripts must include a data availability statement. This statement should provide the following information, where applicable:

- Accession codes, unique identifiers, or web links for publicly available datasets
- A list of figures that have associated raw data
- A description of any restrictions on data availability

Data Availability: Coordinates and structure factors have been deposited in the wwwPDB.
Rat KMO in complex with compound 3 PDB ID 6LKD
Rat KMO in complex with compound 4 PDB ID 6LKE

Field-specific reporting

Please select the one below that is the best fit for your research. If you are not sure, read the appropriate sections before making your selection.

☒ Life sciences ☐ Behavioural & social sciences ☐ Ecological, evolutionary & environmental sciences

For a reference copy of the document with all sections, see nature.com/documents/nr-reporting-summary-flat.pdf

Life sciences study design

All studies must disclose on these points even when the disclosure is negative.

Sample size	No statistical methods were used to predetermine sample sizes
Data exclusions	No data were excluded
Replication	All experiments were confirmed with multiple biological replicates as detailed in Methods or Figure Legends
Randomization	No randomization was performed
Blinding	No blinding is used

Reporting for specific materials, systems and methods

We require information from authors about some types of materials, experimental systems and methods used in many studies. Here, indicate whether each material, system or method listed is relevant to your study. If you are not sure if a list item applies to your research, read the appropriate section before selecting a response.

Materials & experimental systems

n/a	Involved in the study	
☐	☒	Antibodies
☐	☒	Eukaryotic cell lines
☒	☐	Palaeontology
☐	☒	Animals and other organisms
☒	☐	Human research participants
☒	☐	Clinical data

Methods

n/a	Involved in the study	
☒	☐	ChIP-seq
☒	☐	Flow cytometry
☒	☐	MRI-based neuroimaging

Antibodies

Antibodies used	Anti-GST-tag pAb (MBL Code No.PM013), IRdye 800CW anti Rabbit LICOR 926-32211
Validation	All antibodies used in this study are commercial. They are evaluated in Extended Data Fig. 10B<br>Anti-GST-tag pAb (MBL Code No.PM013) There are 4 citations in the manufacturer's website http://ruo.mbl.co.jp/bio/e/dtl/A/?pcd=PM013<br>IRdye 800CW anti Rabbit LICOR 926-32211 There are 95 citations in the manufacturer's website https://www.biocompare.com/9776-Antibodies/245710-Goat-AntiRabbit-IgG-IRDyereg-800CW-Conjugated/

Eukaryotic cell lines

Policy information about cell lines

Cell line source(s)	HEK293 was obtained from ATCC (Cat# CRL-1573)<br>HEK293T was obtained from Thermo Fischer Scientific (Cat HCL4517).<br>Sf9 cells were obtained from Thermo Fischer Scientific (Product No. 12659017).
Authentication	Cell lines were verified by manufacturer's webstite and identity of these cell lines were frequently checked by their morphological features. All cell lines were kept at low passages in order to maintain their identity.
Mycoplasma contamination	All cell lines were tested to be mycoplasma-negative by PCR.
Commonly misidentified lines<br>(See ICLAC register)	No commonly misidentified cell lines are used in this study.

Animals and other organisms

Policy information about studies involving animals; ARRIVE guidelines recommended for reporting animal research

Laboratory animals	Sprague-Dawley rats Crl:CD, Male, 8 weeks of age
Wild animals	The study did not involve wild animals.
Field-collected samples	The study did not involve field-collected samples.
Ethics oversight	All animal experimental procedures were approved by the Institutional Animal Care and Use Committee of Astellas Pharma Inc. Astellas Pharma Inc., Tsukuba Research Center, has been awarded Accreditation Status by The Association for Assessment and Accreditation of Laboratory Animal Care (AAALAC) International.

Note that full information on the approval of the study protocol must also be provided in the manuscript.
